# Event cache: An independent component in working memory

**DOI:** 10.1126/sciadv.adt3063

**Published:** 2025-05-23

**Authors:** Hui Zhou, Jinglan Wu, Jiaofeng Li, Zhihe Pan, Jinying Lu, Mowei Shen, Tengfei Wang, Yuzheng Hu, Zaifeng Gao

**Affiliations:** ^1^ Department of Psychology and Behavioral Sciences, Zhejiang University, Hangzhou 310058, China.; ^2^ The State Key Lab of Brain-Machine Intelligence, Zhejiang University, Hangzhou 310058, China.; ^3^ MOE Frontiers Science Center for Brain Science & Brain-Machine Integration, Zhejiang University, Hangzhou 310058, China.; ^4^ Nanhu Brain-Computer Interface Institute, Hangzhou 311121, China.

## Abstract

Working memory (WM) has been a major focus of cognitive science and neuroscience for the past 50 years. While most WM research has centered on the mechanisms of objects, there has been a lack of investigation into the cognitive and neural mechanisms of events, which are the building blocks of our experience. Using confirmatory factor analysis, psychophysical experiments, and resting-state and task functional magnetic resonance imaging methods, our study demonstrated that events have an independent storage space within WM, named as event cache, with distinct neural correlates compared to object storage in WM. We found the cerebellar network to be the most essential network for event cache, with the left cerebellum Crus I being particularly involved in encoding and maintaining events. Our findings shed critical light on the neuropsychological mechanism of WM by revealing event cache as an independent component of WM and encourage the reconsideration of theoretical models for WM.

## Teaser

The “event cache,” a working memory component reshaping our understanding of event storage and processing, is discovered.

## INTRODUCTION

Working memory (WM) refers to a system that temporarily stores and manipulates a limited set of information for ongoing tasks (*1*–*3*). Although WM capacity is limited, it is essential for high-order cognitions, such as fluid intelligence (*4*), reading comprehension (*5*), planning (*6*), and creative thinking (*7*). WM deficits have been reported in various mental disorders such as Alzheimer’s disease, attention deficit hyperactivity disorder, autism spectrum disorders, and schizophrenia (*8*–*11*). Understanding the mechanisms of WM has substantial educational and clinical implications and has been a key topic in cognitive science and neuroscience over the past 50 years (*12*, *13*).

Different WM theories have been proposed to elucidate how information is stored and manipulated in WM. Two of the most influential models are the multicomponent model and the embedded-processes model (*1*–*3*). The multicomponent model claims that WM consists of four components: central executive (CE), phonological loop, visuospatial sketchpad, and episodic buffer (*2*, *14*). The CE is responsible for coordinating and monitoring ongoing tasks in a top-down manner, while the remaining three are responsible for retaining verbal, visuospatial, and binding information (*14*). Moreover, these distinct components are supported by different neural substrates or networks (*2*). In contrast, the embedded-processes model and its variants suggest that all information is stored in a capacity-limited focus of attention (FoA), which is a subset of information in long-term memory that is temporarily activated (*15*, *16*).

Because of the reductionism routine, where complex information from the external world is simplified into more fundamental components, and past technical limitations in psychological explorations, the main body of WM studies, including theoretical models, has focused on verbal and visual objects and their constituent features (*2*, *17*). However, in daily life, individuals not only process objects and features (e.g., shapes, colors, and motion) but also attend to what happens to them—namely, events. Events represent a distinct psychological construct, separate from objects, and are characterized by their temporal and spatial structures (*18*, *19*). Here, we operationally define an event as a spatiotemporally bound segment with clear initiation and termination points (*20*). While events are typically characterized by spatiotemporal boundaries, not all stimuli with such boundaries qualify as events. A key feature of an event is its perceived coherence—it generally maintains an internally structured and interpretable sequence throughout its duration (*20*), with boundaries that naturally align with its intrinsic meaning. For example, biological movements (BMs) such as running or jumping exhibit coherence and thus constitute events, whereas a sequence of random, unstructured movements—such as dynamic noise—does not. While some theoretical frameworks suggest that static scenarios, such as a glass resting on a table, can be classified as an event because of their functional significance (*21*), the predominant approach in event cognition differentiates static states from dynamic events, emphasizing that events typically involve structured temporal progression. Accordingly, this study focuses on the examination of dynamic events and their cognitive representations.

Events are fundamental units of our experience, shaping the way we connect and separate various occurrences (*19*, *22*). The human brain has evolved to use temporal structures in events to predict incoming information and enable functional behaviors. Consequently, events are considered to be one of the most important classes of entities in daily psychology (*18*, *19*) and should be a critical unit of analysis for most psychological domains, including WM. Recent studies have revealed that continuous information flow from the environment is segmented into events in the WM (*22*–*25*). Existing research on events has been focusing on how an event is perceptually segmented from a continuous information flow and what is the temporal structure of an event (*22*). In addition, an event has been extensively explored from the perspective of episodic memory, according to which the term of “event memory” was created (*22*, *26*). However, neither the psychological construct nor the underlying neural substrates of event processing in WM are well understood. In particular, it remains unknown whether there is a unique cache for events in WM.

Addressing this question is essential, as it touches on the core mechanisms of how the human brain organizes information over time. While both animate actions (e.g., BMs) and nonanimate changes (e.g., physical object movements) are often studied as representative examples of events in the past half-century (*19*, *20*, *22*), to our knowledge, no study has systematically examined whether event storage constitutes a distinct WM component. Filling this gap requires not only robust empirical evidence but also a multidisciplinary approach capable of disentangling complex cognitive constructs.

**Fig. 1.**
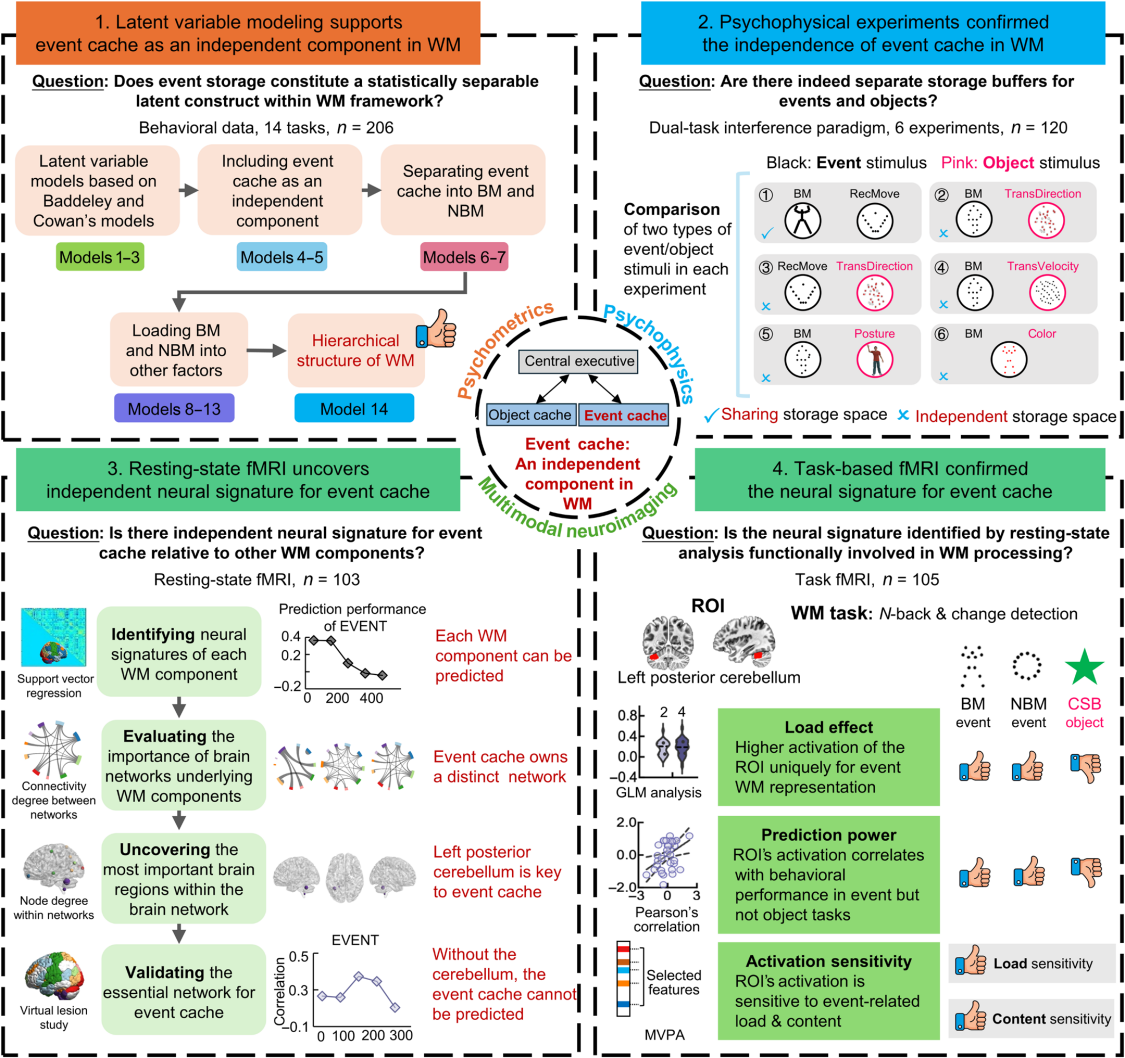
Flowchart of the current study. First, confirmatory factor analysis (CFA) was used to test candidate models on the behavioral data. Subsequently, six dual-task experiments were conducted to validate the independence of event cache from object cache and confirm the model chosen by CFA. Next, support vector regression (SVR) was conducted on resting-state functional magnetic resonance imaging (fMRI) data to identify neural signatures of working memory (WM) components in the optimal model. The most important network for event cache was identified by evaluating the network-wise connectivity degree. The node degree for each brain region in this network was calculated to find regions most contributing to the prediction of the event cache. Subsequently, virtual lesion analysis was done to assess each network’s importance in prediction. Last, region of interest (ROI)-based analysis was performed on task fMRI data to verify the specific functional implications of the event-associated brain regions identified with resting-state data, and multivariate pattern analysis (MVPA) was additionally performed to deeply explore the role that the event-associated brain regions play in event-load and event-content processing. TransDirection, direction of transparent motion; TransVelocity, velocity of transparent motion; CSB, color-shape binding.

To rigorously address this question, we adopt a multimodal integrative approach transcending traditional methodological boundaries. Specifically, four complementary investigative strands provide converging evidence (Fig. 1). First, psychometric validation through confirmatory factor analysis (CFA) of 14 established WM tasks establishes whether event storage constitutes a statistically separable latent construct within the WM framework. Second, six psychophysical experiments using dual-task paradigms were conducted to provide behavioral evidence for the independence of event storage in WM. Third, machine learning analysis of resting-state functional magnetic resonance imaging (fMRI) connectivity patterns identifies neural signatures predictive of event-specific WM capacity. Last, task-based fMRI examines the spatiotemporal dynamics of cortical activation during the event versus object maintenance, aiming to verify the findings derived from resting-state fMRI. This methodological triangulation—spanning psychometrics, psychophysics, and multimodal neuroimaging—enables robust cross-validation of findings while mitigating the limitations inherent to any single methodology.

## RESULTS

### Latent variable modeling indicates event cache as an independent component in WM

#### Descriptive statistics of WM measures used for latent variable modeling

To estimate the latent variable models of WM, we used 14 tasks to tap the different components of WM. There were four event storage tasks [point-light display (PLD) BM, solid BM, rectangular movements (RecMoves), and circular movements], three object storage tasks (colors, shapes, and locations), two binding storage tasks (color-location and color-letter bindings), two CE tasks (anti-saccade and *N*-back), and three other tasks [operation span (Ospan), symmetry span (Sspan), and multiple-object tracking (MOT)]. The two complex span tasks (Ospan and Sspan) (*4*, *27*) have been used frequently to tap the capacity of storing information in the presence of distractions, reflecting the joint function of storage and CE of WM (*28*). The common variance shared by the CE tasks and the two complex span tasks therefore mainly represents CE. The MOT, which has dynamic features without persisting higher-order stability, relies on the visuospatial sketchpad to process moving objects for a short interval (*29*). For both event and object storage tasks, we used a simultaneous-display change detection paradigm, which is a standard approach in the object WM research. An eye-tracking experiment confirmed that participants can extract multiple events by sequentially encoding each dynamic item in this simultaneous-display setting (fig. S1; see Supplementary Text S1.1 to S1.3). A detailed rationale for task selection is provided in the Materials and Methods.

Cowan’s *K* (*30*) was used to estimate the WM capacity for each storage task. The formula of Scholl *et al.* (*31*) was used to estimate the tracked capacity of the MOT, and task accuracy was used to indicate the WM performance for the CE tasks and span tasks. Detailed descriptive statistics for the 14 WM tasks are presented in table S1, and their correlations are presented in table S2. Most measures showed acceptable levels of reliability (≥0.60) and relatively low skewness and kurtosis values (between −2 and 2). The tasks assumed to tap the same WM component showed relatively higher correlations than those of a different WM component, suggesting both the convergent and discriminant validity of these measures. Overall, the WM tasks with strictly controlled experimental settings (the change detection paradigm), appropriate difficulty, good reliability, and validity provided a favorable prerequisite for latent variable modeling.

#### Latent variable modeling based on Baddeley and Cowan’s WM models is suboptimal

We initially evaluated three models according to Baddeley and Cowan’s WM models (fig. S2). It is worth noting that events in the current testing were probed using visual stimuli, and verbal encoding was suppressed; hence, we did not consider the phonological loop in the multicomponent model of WM.

Model 1 was constructed to test whether all tasks could fit Cowan’s embedded-processes model (*15*), which included CE and FoA.According to this model, different types of stimuli are stored in FoA. Therefore, all tasks entailing the storage of visuospatial stimuli, binding, and events were assumed to load on a common FoA latent variable, while other tasks relating to CE loaded on the CE latent variable. Models 2 and 3 were constructed to test how well the tasks fit Baddeley’s multicomponent model (*14*). The only distinction between these two models was that the event storage tasks were loaded on a visuospatial sketchpad (model 2) or episodic buffer (model 3). The fit statistics of all the models are listed in table S3. Models 1 to 3 showed poor fitness according to the comparative fit index (CFI), suggesting that the two well-established models cannot fully account for the underlying structure of WM. In particular, the representations of events are probably not stored in FoA, conventional visuospatial sketchpad, or episodic buffer. It is possible that there is an additional storage space for events.

#### Models with event tasks loading on an independent component are superior

We further tested another set of models considering an independent event cache for holding events in WM (fig. S2). We established model 4 based on Cowan’s model, wherein the FoA was divided into event cache and object cache, with the former being responsible for storing events and the latter for static visual stimuli. Alternatively, we established model 5 according to Baddeley’s model. In model 5, an event cache was introduced in addition to a visuospatial sketchpad and episodic buffer. Both models 4 and 5 showed an acceptable fit to the data (table S3). These results clearly demonstrate that there is an independent event cache underlying the WM structure.

The two types of events in this study have distinct properties. BMs of humans contain rich social information, whereas nonbiological movements (NBMs; i.e., RecMoves and circular movements) of physical shape do not (*32*, *33*). To further explore whether there are two different types of event cache owing to the difference in social semantics, we constructed models 6 and 7 based on models 4 and 5, respectively, by separating event cache into BM and NBM latent variables. Both models were acceptable. However, model 6 showed a better fit to the data than the other three models according to the comparisons of CFI and Akaike’s information criterion (AIC) (table S3). Furthermore, loading either BM or NBM into a visuospatial sketchpad, episodic buffer, or object cache worsened model fitting (see models 8 to 13 in fig. S3), suggesting that events, including BM and NBM, are represented separately from objects in WM.

**Fig. 2.**
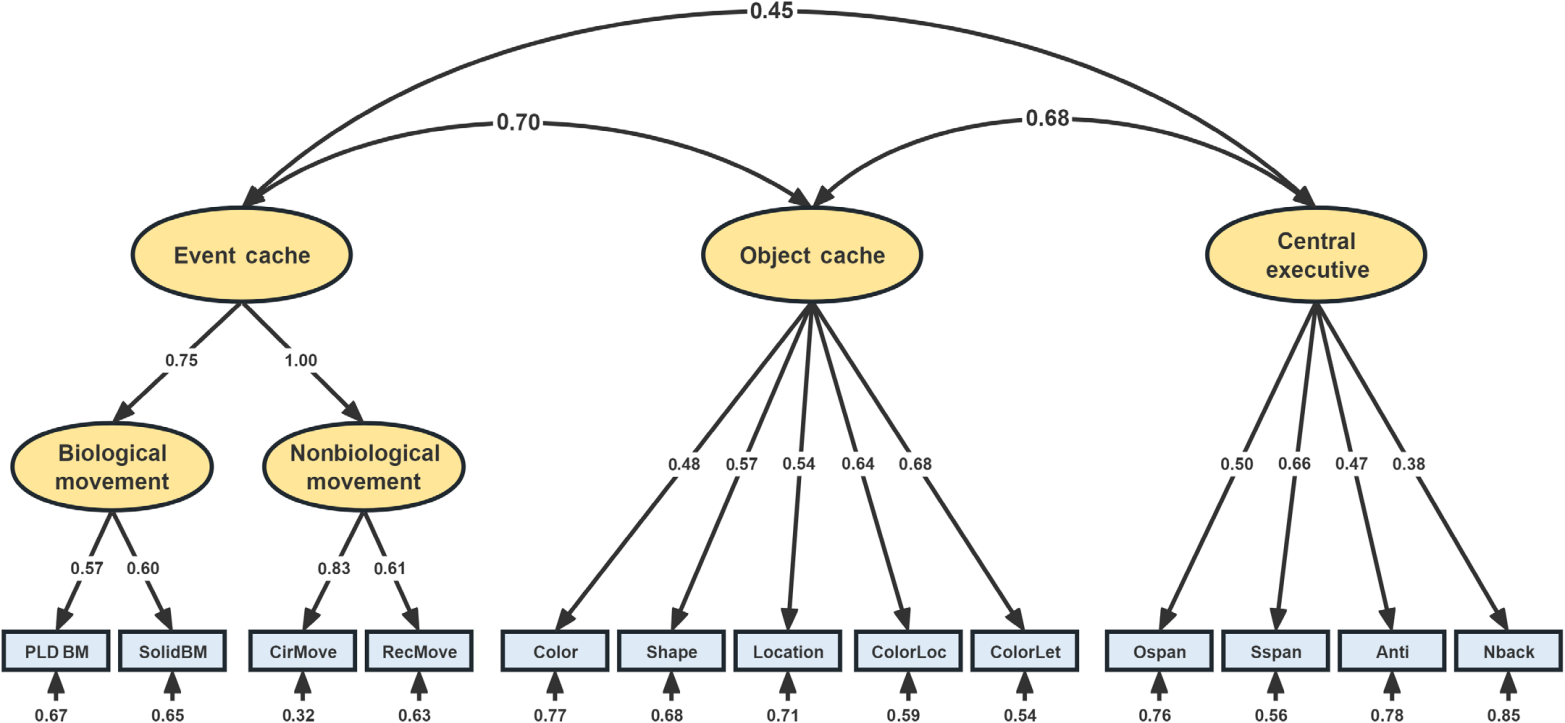
Hierarchical structural model of WM (model 14). SolidBM, solid biological movement; CirMove, circular movement; ColorLoc, color-location binding; ColorLet, color-letter binding; Anti, anti-saccade. The nonbiological movement factor yielded a nonsignificant negative residual variance, which therefore had to be fixed at zero. Asterisks indicate significant paths or loadings (**P < 0.01). Ellipses represent latent variables, and rectangles represent observed indicators. The single-headed arrow points from a latent factor to an observed indicator or from a higher-order factor to lower-order factors, and the double-headed arrow indicates the correlation between latent variables. The single-headed arrow on an observed indicator indicates the error term.

Given that the tasks of BM and NBM both tap the nature of events, we tested whether an underlying common event cache could be captured by a higher-order factor. We constructed model 14 based on model 6 (the best nonhierarchical model), where event cache was conceptualized as a second-order latent variable extracted from BM and NBM factors (Fig. 2). We used the chi-square (χ^2^) difference test to compare these two nested models. According to the result (χ_diff_^2^ = 2.6, *P* = 0.273), there is no significant difference between the two models. On the basis of the principle of model simplicity, we should choose model 14, which was simpler (with greater degrees of freedom), to be the best description of the WM construct.

#### Event cache is not simply driven by the motion features of the event stimuli

It is reasonable to conjecture that event cache differed from object cache in the model, mainly because the to-be-remembered stimuli contained motion feature in the event storage tasks but static in the object storage tasks. To rule out this possibility, we constructed four models in which the MOT was loaded on the latent variable of BM (model a), NBM (model b), object cache (model c), and CE (model d) based on model 14. The fit statistics of the models are presented in table S3. The best model (model c) shows that object cache is responsible for storing moving objects. Therefore, the finding indicates that event cache differing from object cache is not due to the motion feature of task stimuli but rather the intrinsically different mechanisms between event storage and object storage in WM.

In short, our behavioral data suggest that event cache is an independent component of WM, which is distinct from object cache that is responsible for the WM storage of single features and binding information. Moreover, this distinction is not due to the motion feature of the task stimuli. In addition, the averaged WM capacity of event tasks (*K* = 2.96) did not differ from that of object tasks [*K* = 3.00; *t* (205) = −0.837, *P* = 0.404, Cohen’s *d* = −0.058], suggesting that the separation of event and object latent variables is not driven by task difficulty.

### Psychophysical experiments further confirm the independence of event cache in WM

**Fig. 3.**
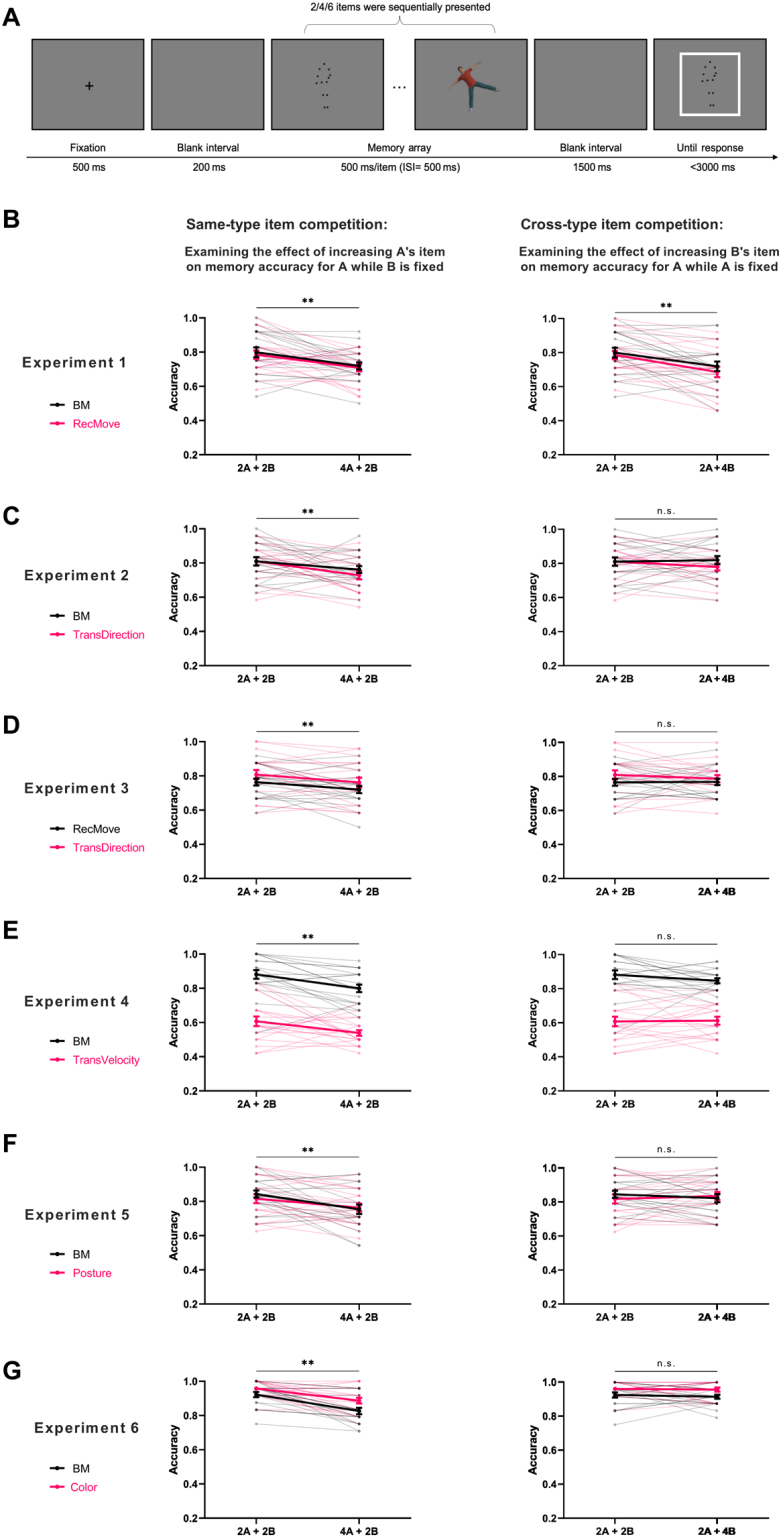
Design of the dual-tasks and behavioral results. (**A**) Schematic illustration of a single trial in the dual-task paradigm. (**B** to **F**) Memory performance for different types of stimuli in experiments 1 to 5, where the memorized items were presented sequentially. The first column shows the results of memory accuracy for type A stimuli under different loads (2 or 4) of A while keeping the load of B fixed at 2. The significant decrease in performance under high-load condition indicates the effectiveness of WM load manipulation. The second column shows the results of memory accuracy for fixed-load materials (type A, load 2) under different loads of the other type of material (type B, load 2 or 4) using the load effect to investigate whether the two types of stimuli compete for the storage capacity. (**G**) Memory performance for experiment 6, where four colorized BMs were displayed simultaneously. In the probe, a black BM or a color square was displayed in the screen center. The other aspects were the same as in experiments 1 to 5. The legend in the figure represents the stimulus used as type A in the *x* axis. The black color indicates the stimulus targeting the event component; the pink color indicates the stimulus targeting the object component. The error bar stands for the standard error of the mean. “**” denotes significant load effect *P* < 0.01, “*” denotes significant load effect 0.01 < *P* < 0.05, and “n.s.” denotes a nonsignificant load effect.

To verify that there are separate storage buffers for events and objects, we conducted six experiments using the dual-task paradigm (Fig. 3A), wherein participants were required to simultaneously memorize two types of randomly presented stimuli (below, we used A and B to denote two distinct types of stimuli). If A and B shared the same storage buffer, they would compete for the limited storage capacity of WM. To be specific, experiments 1 to 5 consisted of five load conditions (2A, 2B, 2A + 2B, 2A + 4B, and 4A + 2B) and experiment 6 contained three conditions (2A + 2B, 2A + 4B, and 4A + 2B), where the numbers indicate how many items are under these conditions. Trials of these conditions were presented randomly, and each type of stimulus was probed with equal probability. Each experiment contained 20 participants, with the sample size determined using the sequential Bayes factor design (*34*). Conditions 2A and 2B served the purpose of introducing lower WM load scenarios and ensuring good WM performance when memorizing two stimuli (accuracy ≥70% for both conditions in all the experiments; see table S4), hence effectively averting the emergence of floor effects under higher-load conditions (i.e., retaining four or six stimuli) in a sequential display. Detailed descriptive statistics of each experiment are presented in table S4.

Two groups of repeated-measures analysis of variance (ANOVA) on memory accuracy were performed. The first group, which was named as same-type-manipulation ANOVA (abbreviated as STM_ANOVA), was used to verify the effectiveness of memory load manipulation by increasing two items that belong to the same type of material. A significant main effect of load was expected in a 2 (material type: A versus B)–by–2 (load: 2 and 4) ANOVA, in which the memory accuracy of A under the conditions of 2A + 2B and 4A + 2B and the memory accuracy of B under 2A + 2B and 2A + 4B were tested. The second group, which was named as cross-type-manipulation ANOVA (abbreviated as CTM_ANOVA), was used to test whether the two types of materials share a common storage in WM. If they do, a significant load effect should be identified by a 2 (material type: A versus B)–by–2 (load: 2 and 4) ANOVA, in which the memory accuracy of A taken from the conditions of 2A + 2B and 2A + 4B and the memory accuracy of B taken from the conditions of 2A + 2B and 4A + 2B were tested.

#### Experiment 1: The storage of BM and NBM events interferes with each other

As the BM and NBM (RecMove) were used to establish the “event” component, we first tested the hypothesis that the representations of these two types of stimuli would interfere with each other. Results of the STM_ANOVA showed that the increase in WM load of the stimulus type to be detected significantly decreased the memory accuracy (main effect of load: *F*_1,19_ = 13.171, *P* = 0.002, $\eta_{p}^{2}$ = 0.409, *BF*_10_ = 18.851, Fig. 3B), indicating that the load manipulation was effective. The main effect of material type (*F*_1,19_ = 0.493, *P* = 0.491, $\eta_{p}^{2}$ = 0.025, *BF*_10_ = 0.321) and the interaction (*F*_1,19_ = 0.009, *P* = 0.926, $\eta_{p}^{2}$ = 4.675 × 10^−4^, *BF*_10_ = 0.314) were nonsignificant.

Meanwhile, results of the CTM_ANOVA showed that the increase in WM load of the stimulus type not to be detected also had a significant load effect on the memory accuracy of the stimulus type being detected (main effect of load: *F*_1,19_ = 22.020, *P* < 0.001, $\eta_{p}^{2}$ = 0.537, *BF*_10_ = 95.945, Fig. 3B), suggesting that BM and RecMove share a common storage space in WM. The main effect of material type (*F*_1,19_ = 1.457, *P* = 0.242, $\eta_{p}^{2}$ = 0.071, *BF*_10_ = 0.498) and the interaction (*F*_1,19_ = 0.265, *P* = 0.612, $\eta_{p}^{2}$ = 0.014, *BF*_10_ = 0.447) were nonsignificant. This result further confirms our CFA result that BM and NBM belong to the same event component.

#### Experiment 2: The storage of BM events and objects (the direction of transparent motion) does not interfere with each other

Then, we tested the possibility that BM and RecMove share one buffer, which may be due to the fact that they both have motion feature instead of the putative event nature. A previous study has shown that the transparent motion is represented in the form of object (*35*). To this end, we replaced the RecMove with transparent motion consisting of dots that continuously move in one direction without beginnings or endings. Participants were required to retain the moving direction of transparent motion.

The STM_ANOVA showed that the increase in WM load of the stimulus type to be detected significantly decreased the memory accuracy (main effect of load: *F*_1,19_ = 8.418, *P* = 0.009, $\eta_{p}^{2}$ = 0.307, *BF*_10_ = 5.040, Fig. 3C), indicating that the load manipulation was effective. The main effect of material type (*F*_1,19_ = 0.541, *P* = 0.471, $\eta_{p}^{2}$ = 0.028, *BF*_10_ = 0.387) and the interaction (*F*_1,19_ = 0.818, *P* = 0.377, $\eta_{p}^{2}$ = 0.041, *BF*_10_ = 0.429) were nonsignificant.

However, results of the CTM_ANOVA showed that the increase in WM load of the stimulus type not to be detected had no significant load effect on the memory accuracy of the stimulus type being detected (main effect of load: *F*_1,19_ = 0.424, *P* = 0.523, $\eta_{p}^{2}$ = 0.022, *BF*_10_ = 0.332, Fig. 3C), suggesting that BM and transparent motion do not share a common storage space. The main effect of material type (*F*_1,19_ = 1.367, *P* = 0.257, $\eta_{p}^{2}$ = 0.067, *BF*_10_ = 0.425) and the interaction (*F*_1,19_ = 1.103, *P* = 0.307, $\eta_{p}^{2}$ = 0.055, *BF*_10_ = 0.655) were nonsignificant. This result echoes with the CFA results showing that the MOT task did not load onto the event component and excludes the motion feature hypothesis of experiment 1.

#### Experiment 3: The storage of NBM events and objects (the direction of transparent motion) does not interfere with each other

To examine whether the noncompetition between BM event and transparent motion could be generalized to another type of event, we replaced BM in experiment 2 with the NBM (i.e., RecMove). The STM_ANOVA results demonstrated that the increase in WM load of the stimulus type to be detected significantly decreased the memory accuracy (main effect of load: *F*_1,19_ = 10.442, *P* = 0.004, $\eta_{p}^{2}$ = 0.354, *BF*_10_ = 3.042, Fig. 3D), indicating that the load manipulation was effective. The main effect of material type (*F*_1,19_ = 2.787, *P* = 0.111, $\eta_{p}^{2}$ = 0.128, *BF*_10_ = 1.101) and the interaction (*F*_1,19_ = 0.004, *P* = 0.952, $\eta_{p}^{2}$ = 1.946 × 10^−4^, *BF*_10_ = 0.281) were nonsignificant.

Critically, results of the CTM_ANOVA showed that the increase in WM load of the stimulus type not to be detected had no significant load effect on memory accuracy (main effect of load: *F*_1,19_ = 0.388, *P* = 0.541, $\eta_{p}^{2}$ = 0.020, *BF*_10_ = 0.326, Fig. 3D), suggesting that RecMoves and transparent motion do not share a common storage space. The main effect of material type (*F*_1,19_ = 2.012, *P* = 0.172, $\eta_{p}^{2}$ = 0.096, *BF*_10_ = 0.690) and the interaction (*F*_1,19_ = 0.563, *P* = 0.462, $\eta_{p}^{2}$ = 0.029, *BF*_10_ = 0.410) were nonsignificant. This result extends the finding of experiment 2, supporting the prediction that RecMoves are stored independently from simple motion features.

#### Experiment 4: The storage of BM events and objects (the velocity of transparent motion) does not interfere with each other

To examine whether the key finding of experiment 2 could be generalized to other motion attributes, we replaced the transparent motion direction task with a transparent motion velocity task. Similarly, the STM_ANOVA demonstrated that the increase in WM load of the stimulus type to be detected significantly decreased the memory accuracy (main effect of load: *F*_1,19_ = 10.842, *P* = 0.004, $\eta_{p}^{2}$ = 0.886, *BF*_10_ = 9.675, Fig. 3E), indicating that the load manipulation was effective. The main effect of material type (*F*_1,19_ = 148.056, *P* < 0.001, $\eta_{p}^{2}$ = 0.886, *BF*_10_ = 5.843 × 10^7^) was significant. The interaction (*F*_1,19_ = 0.136, *P* = 0.717, $\eta_{p}^{2}$ = 0.007, *BF*_10_ = 0.454) was nonsignificant.

Critically, results of the CTM_ANOVA showed that the increase in WM load of the stimulus type not to be detected had no significant load effect on memory accuracy (main effect of load: *F*_1,19_ = 0.496, *P* = 0.490, $\eta_{p}^{2}$ = 0.025, *BF*_10_ = 0.326, Fig. 3E), suggesting that BM and the velocity of transparent motion do not share a common storage space. The main effect of material type (*F*_1,19_ = 146.077, *P* < 0.001, $\eta_{p}^{2}$ = 0.885, *BF*_10_ = 5.436 × 10^7^) was significant. The interaction (*F*_1,19_ = 1.233, *P* = 0.281, $\eta_{p}^{2}$ = 0.061, *BF*_10_ = 0.558) was nonsignificant. This result extends the finding of experiment 2, supporting the prediction that BMs are stored independently from simple motion features.

#### Experiment 5: The storage of BM events and objects (human posture) does not interfere with each other

Our model predicts that any static visual stimuli (i.e., objects) would not compete for the WM capacity with event. In this experiment, static human posture images extracted from BM were used, in contrast to BM. As both BM and human posture contain rich biological information and activate mirror neurons (*36*), the influence of biological information would be controlled in this experiment.

The STM_ANOVA results showed that the increase in WM load of the stimulus type to be detected significantly decreased the memory accuracy (main effect of load: *F*_1,19_ = 14.043, *P* = 0.001, $\eta_{p}^{2}$ = 0.425, *BF*_10_ = 20.885, Fig. 3F), indicating that the load manipulation was effective. The main effect of material type (*F*_1,19_ = 0.192, *P* = 0.666, $\eta_{p}^{2}$ = 0.010, *BF*_10_ = 0.329) and the interaction (*F*_1,19_ = 1.453, *P* = 0.243, $\eta_{p}^{2}$ = 0.071, *BF*_10_ = 0.598) were nonsignificant.

Critically, results of the CTM_ANOVA showed that the increase in WM load of the stimulus type not to be detected had no significant load effect on memory accuracy (main effect of load: *F*_1,19_ = 0.033, *P* = 0.858, $\eta_{p}^{2}$ = 0.002, *BF*_10_ = 0.301, Fig. 3F), suggesting that BM is stored independently from human posture in WM. The main effect of material type (*F*_1,19_ = 0.155, *P* = 0.698, $\eta_{p}^{2}$ = 0.008, *BF*_10_ = 0.327) and the interaction (*F*_1,19_ = 1.641, *P* = 0.216, $\eta_{p}^{2}$ = 0.080, *BF*_10_ = 0.746) were nonsignificant. This result is in line with the prediction that human postures are stored as objects, even though they share similarities with BM.

#### Experiment 6: The storage of BM events and objects (colors) does not interfere with each other

Last, we examined whether the separate retention of events and objects in experiments 2 to 5 stemmed from inherent visual attribute differences (e.g., stimulus offset) among two stimulus categories. We presented four colorized BMs simultaneously, which may contain two distinct BMs and two distinct colors, two distinct BMs and four distinct colors, or four distinct BMs and two distinct colors (fig. S5). Participants were tasked with retaining both sets of information and determining whether the probed BM or color appeared in the memory array.

The STM_ANOVA results demonstrated that the increase in WM load of the stimulus type to be detected significantly decreased the memory accuracy (main effect of load: *F*_1,19_ = 34.943, *P* < 0.001, $\eta_{p}^{2}$ = 0.648, *BF*_10_ = 1870.439, Fig. 3G), indicating that the load manipulation was effective. The main effect of material type (*F*_1,19_ = 17.916, *P* < 0.001, $\eta_{p}^{2}$ = 0.485, *BF*_10_ = 25.399) was significant. The interaction (*F*_1,19_ = 1.114, *P* = 0.304, $\eta_{p}^{2}$ = 0.055, *BF*_10_ = 0.517) was not significant.

Critically, results of the CTM_ANOVA showed that the increase in WM load of the stimulus type not to be detected had no significant load effect on the memory accuracy of the stimulus type being detected (main effect of load: *F*_1,19_ = 0.362, *P* = 0.554, $\eta_{p}^{2}$ = 0.019, *BF*_10_ = 0.331, Fig. 3G), suggesting that BM and color are stored independently in WM. The main effect of material type (*F*_1,19_ = 19.542, *P* < 0.001, $\eta_{p}^{2}$ = 0.507, *BF*_10_ = 21.991) was significant. The interaction (*F*_1,19_ = 0.083, *P* = 0.776, $\eta_{p}^{2}$ = 0.004, *BF*_10_ = 0.342) was nonsignificant. This result echoes with the CFA results showing that the BM and color loaded onto distinct components and excludes the visual attribute difference hypothesis of experiments2 to 5.

Together, the results of these experiments provide strong behavioral evidence for the existence of a separate event cache in WM. To further verify and extend these results, we conducted an extra experiment comparing transparent motion versus human posture (fig. S6). Our results confirmed that the dynamic stimulus (transparent motion in experiment 2) interfered with the static object stimulus (human posture in experiment 5), both belonging to object cache (fig. S7).

### Event cache has independent neural signatures revealed by resting-state fMRI data

#### Support vector regression predicts event cache in addition to classical CE and object components of WM

Based on model 14, we further applied support vector regression (SVR) on resting-state functional connectivity to identify the neural signatures of each WM component [i.e., event cache (EVENT), object cache (OBJECT), and CE]. We computed the functional connectivity matrix for each participant using a brain atlas that consists of 268 areas (nodes) covering the whole brain (*37*), which were assigned to 10 brain networks (*38*) to facilitate network-based analysis. Specifically, the time course of each node was extracted and a Pearson correlation coefficient was calculated between each pair of nodes, which was further transformed to Fisher’s *Z* score to ensure normality. An upper triangular connectivity matrix (35,778 edges) was used in the prediction analyses. On the basis of previous studies that conducted SVR to reveal brain-behavior associations, a prediction model with a feature number of ~200 achieved good performance (*39*, *40*). Thus, we selected several subsets of features with ~200 features to investigate the prediction performance. Specifically, five subsets of features ranking from the top 1 to 9‰, with an increase step of 2‰, were used to predict the individual differences in WM components (with feature numbers ranging from 35 to 322, more subsets were also tested; see fig. S8).

**Fig. 4.**
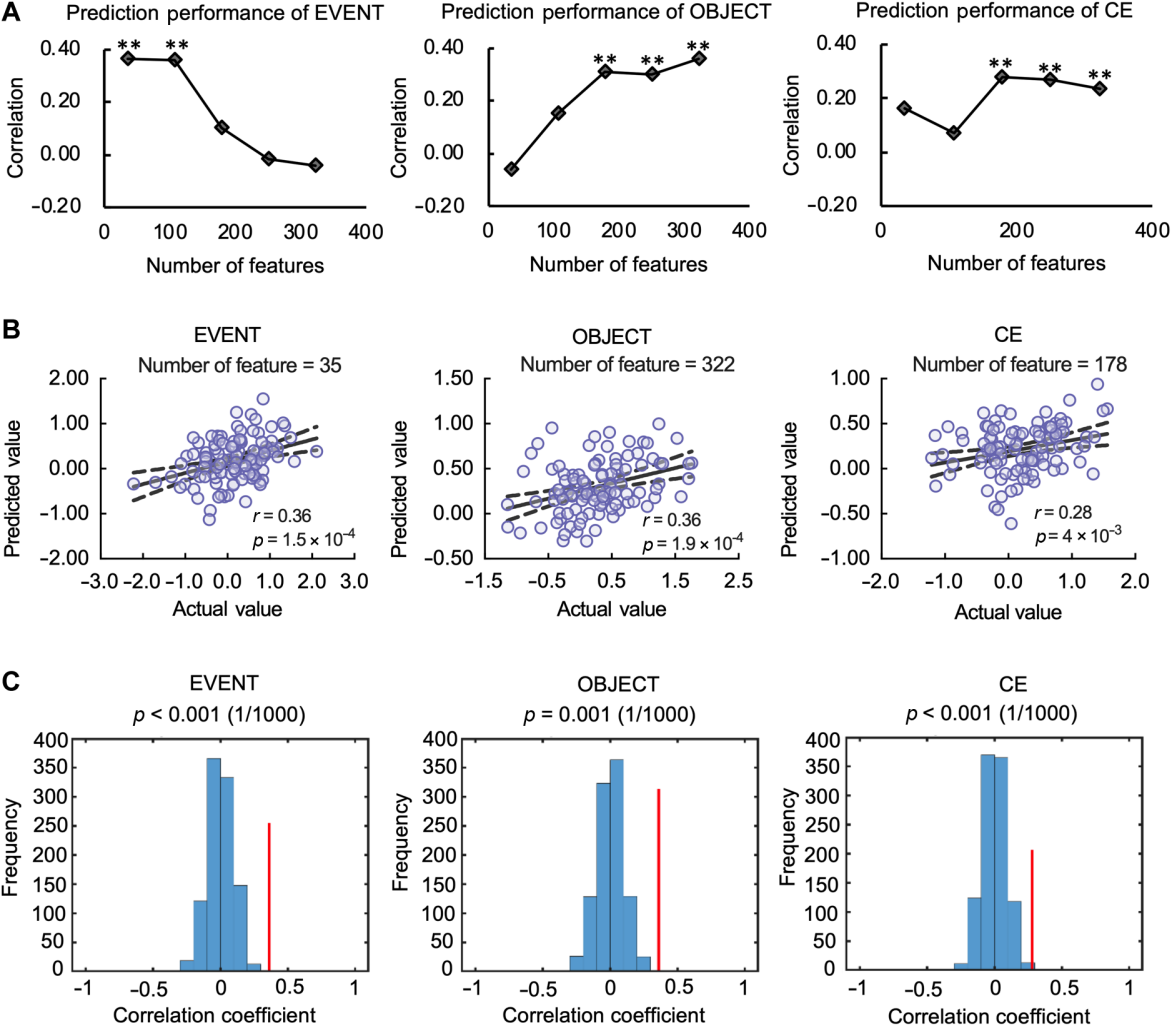
Prediction performance of support vector regression (SVR) models and the correlation between predicted and actual values. (**A**) Prediction performance of the SVR model for each threshold. Note that “**” indicates that the permuted *P* value was considered significant after FDR correction for 15 comparisons (5 sets of feature thresholds × 3 WM components). (**B**) Scatter plots show significant positive correlation between predicted and actual values. The dash line indicates 95% confidence interval. (**C**) Bar plots show the permutation results, with the red line indicating the location of the correlation coefficient between actual and predicted WM scores.

Prediction analyses revealed that all three WM components could be predicted by resting-state functional connectivity patterns (Fig. 4, A and B, and fig. S8). In particular, the EVENT component could be predicted by a relatively small set of edges (with thresholds of 1 and 3‰). With a threshold of 1‰, the prediction model achieved the best performance, and the predicted EVENT scores were positively correlated with the actual values (*r* = 0.36, permutation *P* < 0.001; Fig. 4, B and C). The model for OBJECT prediction achieved the best performance under the threshold of 9‰, and the predicted OBJECT scores were positively correlated with the actual values (*r* = 0.36, permutation *P* = 0.001; Fig. 4, B and C). For the CE component, the prediction model could successfully predict individuals’ CE scores under thresholds of 5, 7, and 9‰, and the model performed the best under the threshold of 5‰. The correlation between predicted and actual values was significant (*r* = 0.28, permutation *P* < 0.001, Fig. 4, B and C). The characteristics of the selected features in the prediction models are visualized under the corresponding thresholds in the “Event WM capacity can be predicted by resting-state brain networks distinct from those of other WM components” and “Brain regions predicting event WM capacity are different from those for other WM components” sections.

#### Event WM capacity can be predicted by resting-state brain networks distinct from those of other WM components

**Fig. 5.**
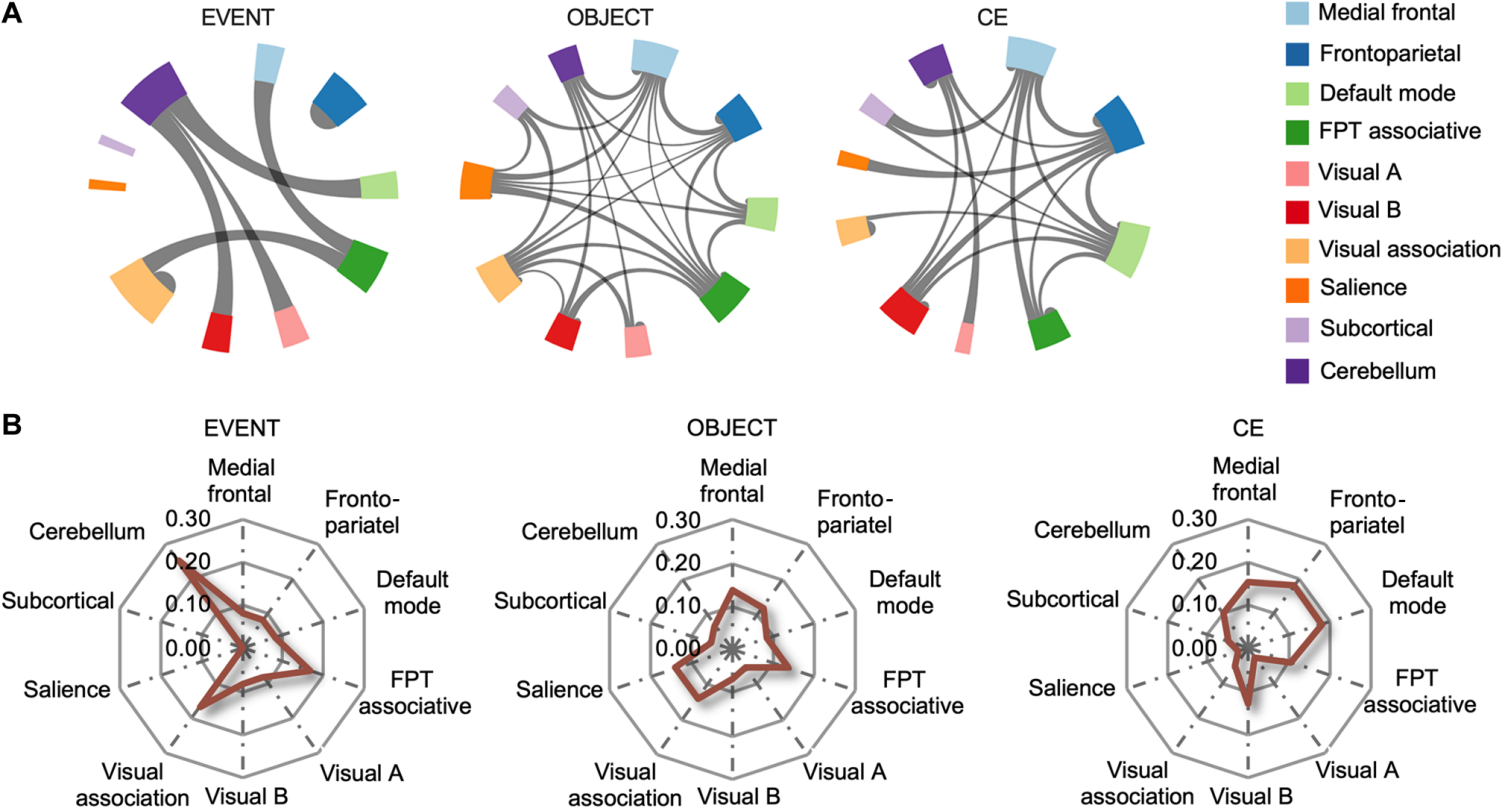
Chord charts of selected features in three prediction models and the relative importance of brain networks for each component. (**A**) Chord charts of between-network Edges show distinct neural signatures for the three WM components. The between-network Edge was the sum of feature weights between nodes that constitute two networks, and the thickness of lines within a chart represents the strength of between-network Edge. (**B**) Radar graphs show successful prediction of the three WM components’ weighting on different networks. FPT associative, frontal-parietal-temporal associative.

To evaluate the importance of different brain networks in the prediction of these three WM components, the 268 nodes were grouped into 10 networks (Fig. 5 and table S5) (*38*), and the edges selected as features in the prediction models were plotted (Fig. 5A). As different features were selected in each iteration, only the features selected in all iterations (103) were considered, and the absolute weights of these features were summed to represent the connectivity between networks (Fig. 5A) with the calculation formula as follows: ${\text{Edge}}_{\mathrm{IJ}}=\sum_{i\in I,j\in J}|\;{\text{edge}}_{\mathrm{ij}}\;|$. Capital letters “*I*” and “*J*” refer to network *I* and network *J*, and “*Edge*” refers to the connectivity strength between two networks. Lowercase letters “*i*” and “*j*” refer to node *i* and node *j*, and “*edge*” refers to the feature weight between the two nodes. To quantitatively illustrate the relative contribution of each network, the relative degree (*RD*) of each network was calculated. The degree of each network (number of edges belonging to a network) was normalized by the total number of existing network degrees using the following formula: ${\text{RD}}_{I}=\frac{\sum_{J}\text{binarize}\left({\text{Edge}}_{\mathrm{IJ}}\right)}{\sum_{I}\sum_{J}\text{binarize}({\text{Edge}}_{\mathrm{IJ}})}$. The capital letters refer to networks, and the “binarize” function transfers a nonzero value to a 1 and keeps a zero value unchanged. The *RD* values are shown in the radar graphs (Fig. 5B). According to the *RD* values (Fig. 5B and table S6), the cerebellum network had the highest *RD* among the 10 networks in the EVENT prediction model and was considered the most important network for predicting the EVENT component, whereas the medial frontal, frontal-parietal-temporal associative, salience, and visual association networks were the top four networks with similar *RD* values for OBJECT prediction. The frontoparietal network and the default mode network played the most important roles in predicting the CE component.

#### Brain regions predicting event WM capacity are different from those for other WM components

Although we identified the most important networks for each WM component, which brain regions within these networks contribute the most remained unclear. To this end, we calculated the degree of nodes (also referred to as node degree) within these networks and ranked nodes according to their degrees to identify the most important nodes within each network (fig. S9 and table S7; for the node degree of all networks, please refer to fig. S10). The results displayed that the node with the largest degree was in the left posterior cerebellum, mainly in Crus I for the EVENT component. As for the OBJECT component, the nodes consisting of the left middle/superior frontal gyrus, right middle/superior temporal gyrus, left inferior/middle occipital gyrus, right postcentral gyrus, superior parietal lobule, and middle cingulate cortex contribute the most in prediction. In contrast, for the CE component, the right middle frontal gyrus within the frontoparietal network and the node covering the left middle occipital gyrus, middle temporal gyrus, angular gyrus, and precuneus within the default mode network had the largest degree.

#### Virtual lesion study on resting-state fMRI data shows that the cerebellar network plays a critical role in predicting event WM

**Fig. 6.**
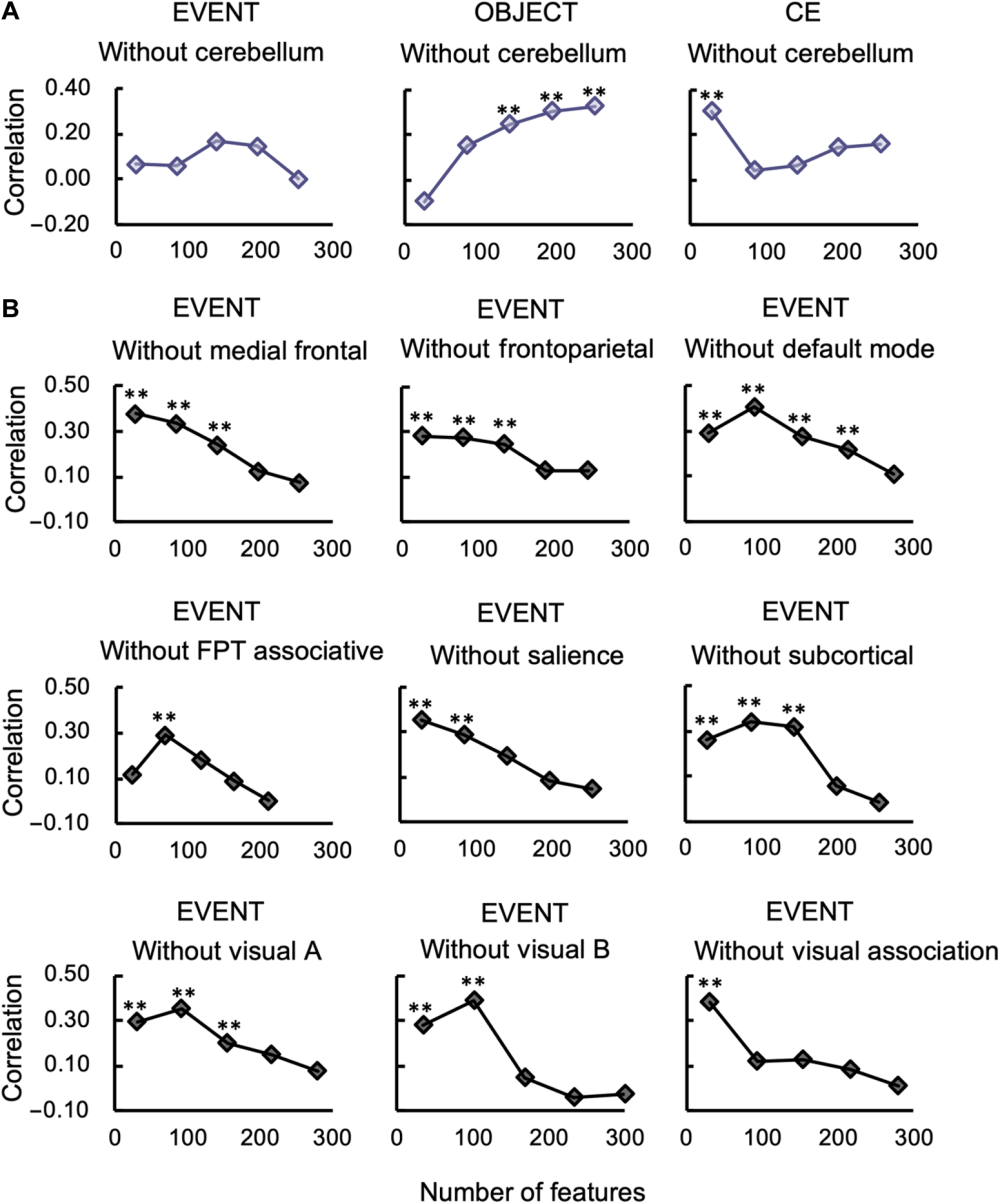
Prediction performance after removing one brain network. (**A**) Prediction performance of EVENT, CE, and OBJECT components after removing the cerebellar network. CE and OBJECT components could still be predicted, but the EVENT component could not be predicted after the cerebellar network was removed. Note that “**” indicates that the permuted *P* value was considered significant after FDR correction for 15 comparisons (5 sets of feature thresholds × 3 WM components). (**B**) The EVENT component could be predicted after removing any of the other nine networks. Note that “**” indicates that the permuted *P* value, was considered significant after FDR correction for 45 comparisons (5 sets of feature thresholds × 9 networks).

To further validate the essential role of the cerebellar network for the EVENT component, the prediction procedure was performed after removing nodes belonging to the cerebellar network (*41*). Permutation tests were performed to confirm the significance, and the false discovery rate (FDR) method was applied to correct for multiple comparisons. The results showed that without the cerebellum, the EVENT component could not be successfully predicted (Fig. 6A), whereas the EVENT component could still be predicted when removing any other network (Fig. 6B). To further investigate whether the role of the cerebellar network is specific for EVENT prediction, we also performed virtual lesion analyses for CE and OBJECT, which showed that without the cerebellar network, CE and OBJECT could still be predicted (Fig. 6A), indicating that the cerebellar network was only essential in predicting the EVENT component. In addition, no single network played a decisive role in predicting OBJECT because the OBJECT component could be successfully predicted regardless of which network was removed (fig. S11). For the CE component, the prediction model failed when the frontoparietal network, medial frontal network, or visual B network was removed (fig. S12).

### The key region of the cerebellar network identified from resting-state fMRI prediction analysis is activated specifically by event tasks

#### Region of interest–based analysis on task fMRI data reveals load-dependent activation of the left posterior cerebellum in event WM representations

**Fig. 7.**
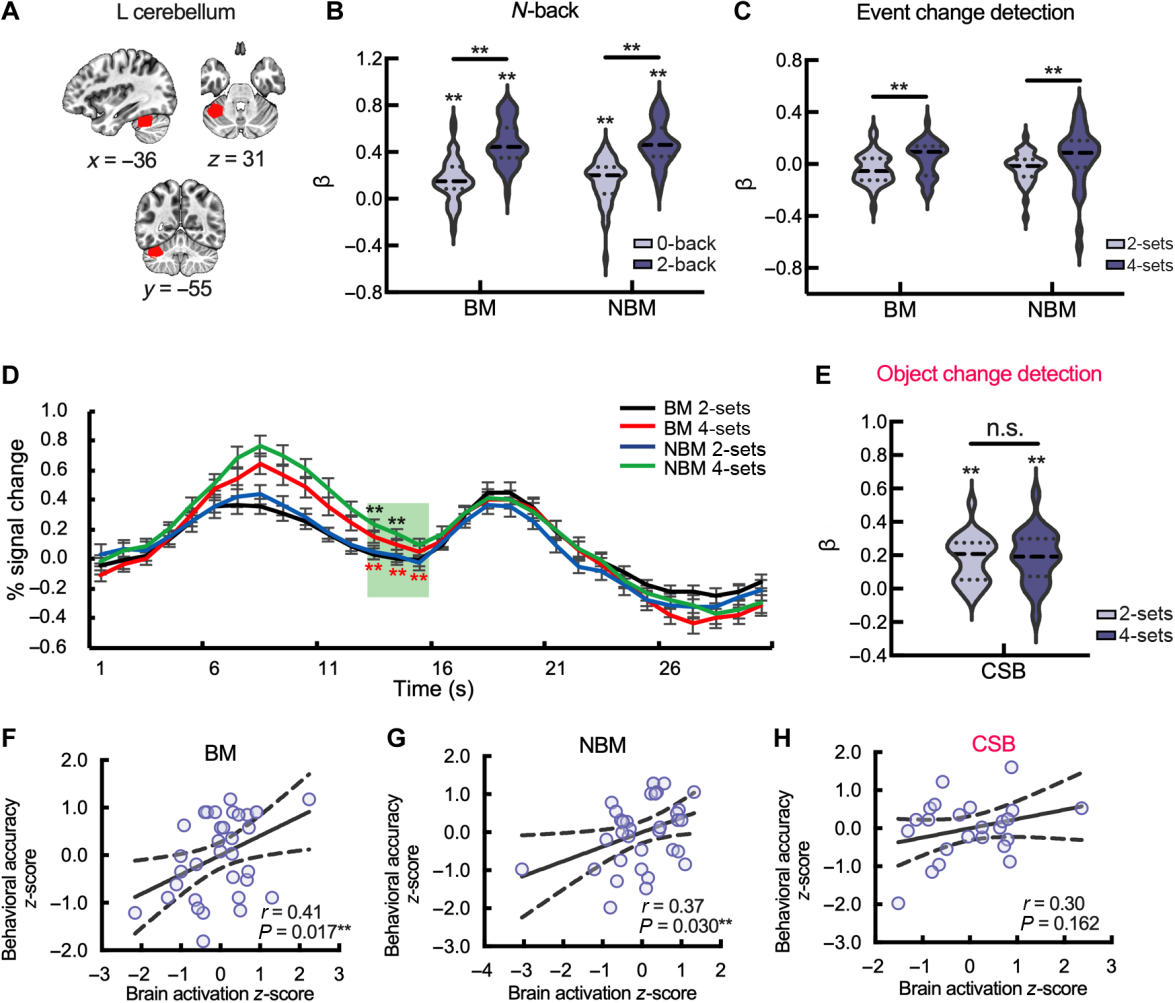
Region of interest (ROI) analyses for WM tasks. (**A**) Anatomical location of the cerebellar node. (**B**) The cerebellar node was significantly activated in the *N*-back task in a load-dependent manner under both BM and NBM conditions. (**C**) The cerebellar node showed similar load-dependent activation (4-sets > 2-sets) during the delay phase of BM and NBM conditions on the event change detection task. The horizontal line in the middle of the violin plots depicts the median, and the lines above and below depict 25th and 75th quartiles. (**D**) Time course–based analysis revealed significant load-dependent activation during the delay phase under both BM and NBM conditions on the event change detection task. The black stars represent the significant load effect under BM conditions, whereas the red stars represent the significant load effect under NBM conditions. The error bar represents the standard error of the mean. (**E**) The cerebellar node was significantly activated during the delay period, but no load-dependent pattern was found on the object (color-shape binding) change detection task. (**F**) The WM performance of BM condition and (**G**) that of NBM condition were significantly correlated with the responses of the left cerebellar node during the delay period. (**H**) The performance of the object task was not significantly correlated with the cerebellar response during the delay period of this task. The dash line in the correlation plot indicates 95% confidence interval. The pink color indicates the stimulus targeting the object component. Note that (A) to (E) illustrate the WM load effect on the cerebellar ROI, and “**” indicates that the *P* value was considered significant after FDR correction for 21 comparisons (10 one-sample *t* tests and 11 paired *t* tests); (F) to (H) depict the brain-behavior correlations, and “**” indicates that the *P* value was considered significant after FDR correction for three comparisons.

Both the prediction analyses and virtual lesion analyses indicated that the resting-state connectivity of the cerebellum played an important role in predicting the EVENT component. In addition, the assessment of nodal contribution to the prediction indicated that the node of the left posterior cerebellum Crus I was the most stable feature for prediction (fig. S9). However, whether this region, identified by resting-state connectivity analysis, is functionally involved in WM processing is unclear. To address this issue, we further examined the activations (i.e., β values) of this cerebellar node (Fig. 7A) using general linear modeling (GLM) analysis on an event *N*-back and a change detection fMRI task.

The behavioral results demonstrated that accuracies of all task conditions were above 0.6 and the accuracy of high-load condition was significantly lower than that of low-load condition (table S8). Brain activation results suggested that Crus I in the left posterior cerebellum was significantly activated in the *N*-back task (both at low and high loads for BM/NBM conditions; Fig. 7B and table S9). Furthermore, significantly higher activation for the high-load condition (2-back) relative to the low-load condition (0-back) was found for both BM and NBM conditions (table S9). In the event change detection task, a significantly higher activation in high load (4-sets) relative to the low load (2-sets) during the delay phase was found under both BM and NBM conditions (table S9 and Fig. 7C). A time-series analysis also confirmed significantly higher activations for the high-load condition (4-sets) relative to the low-load condition (2-sets) during the delay phase under both BM and NBM conditions (Fig. 7D and table S10).

To examine the specificity of the cerebellum region for event cache, we further included an object-based binding change detection task (color-shape binding; fig. S13). This task was selected because the loadings of the binding tasks on the object cache factor were higher than the other object tasks in model 14. The behavioral results revealed that accuracies of all task conditions were above 0.6 and the accuracy of high-load condition was significantly lower than that of low-load condition (table S8). We extracted the β value of the left cerebellum region from the delay period. The results showed that the left cerebellum was significantly activated (both at low and high loads for delay period; Fig. 7E and table S9). However, the load effect (4-sets versus 2-sets) was not significant during the delay period (Fig. 7E and table S9).

#### The activation of the left posterior cerebellum correlates with behavioral performance in event tasks but not object tasks

We further examined the relationship between brain response (the average of *z*-transformed activation for two WM loads) and behavioral performance (the average of *z*-transformed accuracies for two WM loads) on the WM storage tasks (i.e., change detection task). The results showed significant correlations between the cerebellum response during the delay period and performance in the two event tasks (BM: *r* = 0.41, *P* = 0.017, Fig. 7F; NBM: *r* = 0.37, *P* = 0.030, Fig. 7G). However, for the color-shape binding task, no significant correlations were found between cerebellum activation during the delay period and accuracy (Fig. 7H).

In short, using two different event stimulus materials with two different WM paradigms, our data showed that the cerebellar node with the highest predictive power was consistently activated in a load-dependent manner during WM tasks requiring the involvement of the event component, and the cerebellum activation tracks with the WM ability, whereas such a relationship is nonsignificant in the object-based binding task, indicating that the left cerebellum was specific for event cache.

#### The left posterior cerebellum distinguishes event from object processing and tracks WM load in event tasks

Last, to further verify the specific role of the cerebellar region of interest (ROI) in event processing, we conducted multivariate pattern analyses (MVPAs) and found that a classifier trained on activation values from BM low/high loads could distinguish between NBM low and high loads but not between object low and high loads. In addition, a classifier trained on the event (BM) and object conditions could distinguish between these two categories but not between BM and NBM conditions. These results remained consistent when the NBM condition was used as the training set. These results indicate that the activity of this cerebellum region not only tracks load manipulation in the event tasks but also processes event-specific content information. For details in the methodology and results of MVPA, please refer to Supplementary Text S2 (figs. S14 to 16).

## DISCUSSION

### CEO model of WM

**Fig. 8.**
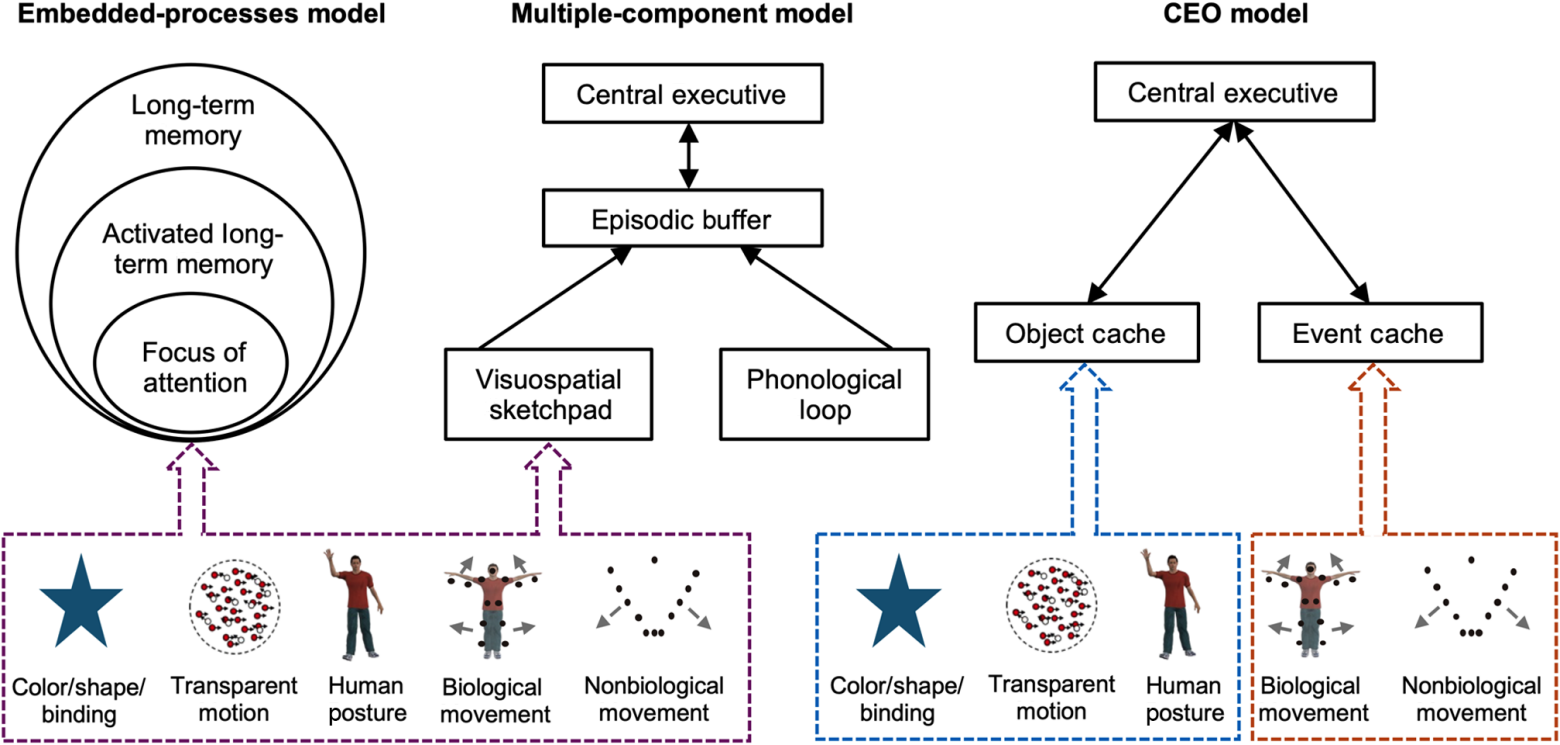
Illustrations of different working memory models with distinct predictions. Both the embedded-processes model and multiple-component model predict that BM and NBM share one storage buffer with objects (including the constituent attributes), while the CEO model predicts that BM and NBM adopt a separate representation format, resulting in independent storage distinct from objects.

By using BM and NBM as the representative stimuli of events in daily life, our study offers evidence supporting an independent storage component (i.e., event cache) in WM. Event cache, as a psychological construct, is distinct from the WM storage space holding objects and related features (i.e., object cache in the current study; Fig. 8). The independence of event cache is further confirmed by psychophysical experiments with dual-task paradigm. Moreover, SVR prediction analyses based on resting-state functional connectivity successfully predicted event cache, object cache, and CE components with distinct patterns. Critically, the cerebellar network emerged as the key component of the event cache, with the left cerebellum Crus I exhibiting the highest node degree in the prediction model. Its activation during event tasks correlated with behavioral performance and was sensitive to event-related load and content. These results provided convergent evidence from behavioral and neural data to support event cache as an independent component of WM.

On the basis of current empirical insights, we have cautiously introduced a model of WM, termed the CEO (central executive-event cache-object cache) model, as a framework to elucidate the conceptual architecture of WM (Fig. 8). In this model, CE assumes the role of overseeing and directing attention across multiple concurrent tasks, while two subordinate components, object cache and event cache, are entrusted with the maintenance of objects (including their constituent attributes) and events, respectively. Object cache and event cache own distinct neural substrates and cognitive processing, leading to resource competition within cache yet no competition between caches. Diverging from the embedded-processes model and the multiple-component model, the CEO model distinctively captures the discrete storage of objects and events. Below, we will discuss the implications of the components embedded in this model.

### Beyond event perception and event memory: Event in WM

The present study provides fresh insights by revealing the existence of a distinct storage space and neural mechanisms dedicated to the representation of events in WM, thereby addressing a crucial gap in our understanding of how the brain processes events. While prior research has predominantly focused on event processing within the domains of perception and episodic memory (*19*, *22*, *26*), the investigation conducted here extends beyond these boundaries. Event perception research has delved into the segmentation of continuous experiences into discrete events, as well as the characterization of these events (*20*, *42*). In parallel, studies concerning event memory have probed the formation, consolidation, and subsequent retrieval of event memory in the brain (*43*, *44*). Notably, the interplay between event segmentation and event memory has been subject to exploration (*22*, *26*). Intriguingly, these inquiries suggest that WM acts as a temporary repository for the products of event segmentation derived from perception, which eventually traverse into episodic memory and undergo intricate processing orchestrated by the hippocampus and prefrontal cortex (*22*). Existing event-related investigations have alluded to the retention of segmented events within a multimodal episodic buffer (*19*). Nevertheless, despite these insights, the precise nature of the storage location for these postsegmentation products within WM remains uncharted territory, marking a notable gap in the existing literature.

Our findings suggest that event cache is responsible for the storage space for the product of event segmentation. Although all stimuli were visually presented in the tasks, we found that visual events had an independent storage space in WM in addition to object cache. Unexpectedly, our investigation did not yield supportive evidence for the presence of an independent episodic buffer in WM. This underscores the likelihood of visual events finding their storage within event cache in WM. It is important to note that our current inquiry represents an initial stride toward unveiling a dedicated buffer for event representation in WM.

### Implications of event cache as an independent psychological construct of WM

The establishment of an independent event component in WM offers critical insights into the development of WM theories. First, while the identification of an event cache supports Baddeley’s speculation (*2*) that there might be an independent store holding human movements in visual WM, we should not grant human movements a special status in WM. Instead, BM and NBM share one buffer, and we need to take human movements as a representative of event representation, which is distinct from object and related features. Although we have known much about WM mechanisms of objects in the past 40 years, the event representation has long been overlooked in the exploration of WM studies. Our study strongly suggests the necessity and importance of extending the scope of WM exploration from objects to events. For example, it remains unclear how events are rehearsed in WM and how events in WM guide external attention.

Second, integrating event cache into the model of WM offers a representation-based explanation to a set of previous empirical findings that human movements are stored independently from colors, shapes, locations, and binding representations (*45*–*47*). Particularly, previous WM studies exploring the storage of human movement have implicitly or explicitly taken human movement as a special stimulus containing rich social information in the environment or as a special form of spatiotemporal information. They argue that human movements own an independent social WM buffer (*32*, *48*) or correspond to a core knowledge system in visual WM (*46*). The current study did not support either view. Our comprehensive evidence from CFA, multimodal neuroimaging, and psychophysical experiments collectively demonstrates that human movements share the same event cache with NBMs, which have a lack of social information, yet were stored independently from human postures.

Third, the differentiation between events and motions suggests that motion features alone are not sufficient to define an event. The MOT task, in which the spatial location of objects needs to be dynamically updated in WM, was found to load on object cache, which aligns with a previous study (*29*). When dealing with moving stimuli that lack clear beginnings and endings, participants did not categorize them as events. They were able to hold both event stimuli (such as human movements or RecMoves) and transparent motion stimuli in WM without capacity competition (experiments 2 to 5). However, there was storage competition between static posture stimuli and transparent motion stimuli (experiment S1). Collectively, these findings suggest that the embedded perceived coherence over time is more critical for defining an event.

Last, event cache may be a hierarchical construct consisting of two subcomponents: BM and NBM. This further dissociation is essentially congruent with recent findings that only WM of BM has an intimate relationship with empathy and theory of mind (*32*, *33*) and exhibited certain distinct neural substrates from NBM (*49*, *50*). From this perspective, the previous claim of social WM indexed by human movements tapped a subcomponent (or social component) of event cache.

### Both visual features and bindings are retained in object cache

Our best model supports the existence of an object cache that is mainly responsible for the storage of visual objects and their constituent features. In contrast to our object cache, Baddeley’s model suggests that the information of bindings and their constituent features are stored in episodic buffer and visuospatial sketchpad, respectively. The episodic buffer was initially assumed to actively bind information from different sources into one episode with the help of CE (*14*). However, few studies have directly examined whether an independent component exists to handle binding (*51*, *52*). Our findings suggest that although the to-be-remembered representations in the binding and feature tasks are different, they are processed by the same object cache and hence may share similar mechanisms. Recent empirical findings suggest that object-based attention plays a key role in retaining both bindings and single-featured objects in WM (*35*, *53*). Therefore, we argue that episodic buffer as an independent buffer for handling bindings should be reconsidered. Note that although color-letter binding theoretically tapped the binding between visual and verbal codes, participants may process the letter both visually and verbally in a short maintenance period (*54*); hence, color-letter bindings in our setting have visual-related representations. Because all bindings in our study contained visual information, future studies need to test the generalization of the current finding using bindings of other modalities (e.g., auditory).

### Event cache, object cache, and CE have distinct neural correlates

The predictions of WM components from resting-state fMRI data suggest that the event cache has a unique neural signature, with the cerebellar network playing a critical role. Traditionally, the cerebellum is considered important for motor coordination and learning (*55*, *56*). However, accumulating evidence supports its involvement in various high-level cognitive functions, including WM (*57*). Evidence from lesion studies has shown that deficits of cerebellum regions or inhibition of cerebellar neuronal excitability significantly reduces the performance of both verbal and visual WM (*58*–*60*). Our task fMRI data showed that the left Crus I of the cerebellum displayed significant activation in response to both BM and NBM. Notably, a positive correlation between Crus I activation and behavioral performance was found in event storage tasks but not in object storage tasks, highlighting its specific role in event WM. Lesion studies have shown that patients with cerebellar damage struggle to detect object movement sequences (a type of event) (*61*, *62*), and the posterior part (especially in Crus I and II) was found to be closely associated with the ability to reconstruct action sequences in a chronological order (*62*, *63*). According to a hierarchical processing perspective of WM (*17*, *64*, *65*), it could be speculated that the cerebellum is involved in constructing high-order representations (referred to as events in our study) of what happened to person(s) or object(s) from dynamic stimuli. Our MVPA results demonstrated that the activation of this cerebellum region not only differentiates event WM from object WM but also can track event WM load. Overall, these convergent empirical neural imaging findings indicate that the cerebellum Crus I may serve as one of the primary neural underpinnings of event cache.

While the left Crus I was examined in detail in the current study, we also recognize that the left Crus I does not function alone to process events. Instead, our data show that the cerebellar connectivity with networks such as the default mode and visual networks also contributes to event cache prediction. This suggests that when individuals conceptualize movements as personally meaningful events, an integration between visual input and intrinsic self-referential processing mechanisms was also required. The primary objective of our task-based fMRI analysis was to uncover the distinct neural bases of event cache and object cache, providing evidence that event cache should be considered as an independent component from object cache. Future studies could build on our findings by using whole-brain or search-light methods to investigate the involvement of broader brain regions in the processing of event WM, thus extending our understanding of the neural basis of event WM.

The neural correlates of CE and object cache revealed by the prediction analysis in the current study are highly consistent with previous studies. Our finding that no single brain network dominates object maintenance aligns with the distributed nature of the cortical regions involved in the maintenance of object features (*17*). As for the CE component, we found that the frontoparietal network and default mode network contributed most to its prediction, with their connectivity also playing a significant role. These results align with previous studies showing the frontoparietal network as the core substrate for tasks requiring executive control (*65*, *66*) and the strength of the functional interaction between these networks correlating with performance on the *N*-back task (*67*), suggesting that the resting-state connectivity–based prediction method can recapture these neural pathways reported by studies that only examined a few sets of connections. These results demonstrate the feasibility of identifying important neural correlates of the event cache and object cache components using the same approach.

### Limitations and future directions

Recognizing the inherent limitations of a single study in covering all event materials, our research builds on the evolutionary history of the event concept and related studies (*18*, *19*, *22*). We selected BM and NBM—two of the most commonly used materials in event research—to investigate the independent storage of events in WM. Our findings provide preliminary evidence for distinct storage spaces for events in WM. However, these results should be considered preliminary because of the limited scope of event materials used, leaving the broader generalizability of the proposed event cache and its functional sensitivity to be explored in future research.

For instance, while our study suggests the presence of a single event cache for various event types (e.g., visual, verbal, and multimodal), the possibility of modality-specific buffers cannot be ruled out. Given the potential relevance of a multimodal event cache, future research could examine how WM handles different types of stimuli beyond the visual modality, such as auditory or verbal events. Furthermore, our study exclusively focused on events perceived from external stimuli. We did not investigate events based on internal mental simulations, such as imagining an event triggered by an auditory cue. Future studies should explore this type of event to better understand how WM processes both externally perceived and internally generated events, which may involve distinct cognitive mechanisms and neural substrates. In addition, our sample consisted of relatively homogeneous young adult participants. The generalizability of our findings across different age groups should be further tested.

It is worth noting that our use of fine-grained tokens for both events and objects aligns with previous studies on the storage spaces of visual objects (*4*, *15*, *46*, *51*, *52*). However, event segmentation studies have unveiled a hierarchical relationship between coarser-grained and finer-grained events, with multiple fine-grained events collectively constituting a coarse-grained event (*20*, *22*, *42*). Future research should investigate how WM processes both coarse-grained and fine-grained events, especially considering that the encoding of such events may involve distinct cognitive strategies. In addition, we used a change detection paradigm, which is commonly used in object cache explorations but differs from the paradigms traditionally used in event research. While this approach has its strengths, future studies should examine whether the storage of events in WM is modulated by task type, as different tasks might tap into different aspects of WM storage and retrieval.

In summary, the event cache proposed in our study paves the way for exploring the multifaceted nature of WM. Further research addressing these limitations and exploring the functional specificity of event storage across populations and modalities will advance our understanding of cognitive flexibility and capacity in various contexts.

## MATERIALS AND METHODS

### Participants

A total of 208 participants (150 females and 58 males) was recruited from the Zhejiang University and Hangzhou Normal University for the CFA. Two participants were excluded because of incomplete data. The final sample that completed behavioral tests (including 7 questionnaires and 14 WM tasks) consisted of 206 participants (148 females and 58 males) aged 17 to 25 years (*M* = 20.4, *SD* = 1.4).

For the psychophysical experiments, 120 participants were recruited through online advertisement [experiment 1, 12 females and 8 males aged 18 to 26 years (*M* = 21.4, *SD* = 2.4); experiment 2, 14 females and 6 males aged 18 to 24 years (*M* = 21.3, *SD* = 1.7); experiment 3, 14 females and 6 males aged 18 to 22 years (*M* = 19.5, SD = 1.2); experiment 4, 10 females and 10 males aged 18 to 29 years (*M* = 21.9, *SD* = 3.0); experiment 5, 16 females and 4 males aged 18 to 48 years (*M* = 22.7, *SD* = 6.3); experiment 6, 8 females and 12 males aged 17 to 27 years (*M* = 21.7, *SD* = 2.7)]. The sample size of the psychophysical experiments was determined on the basis of the sequential Bayes factor design (*34*). The Bayes factor (*BF*_10_) value indicates the ratio of the likelihood of supporting an alternative versus a null hypothesis. Following the sequential Bayes factor design, the *BF*_10_ was repeatedly calculated after reaching the minimum sample size. If the predetermined evidence threshold was not reached, more participants were recruited until the Bayes factors reached this threshold. The minimum sample size was set to 20 before data collection. The threshold for stopping sampling was set to *BF*_10_ > 3 (moderate evidence for *H*_1_) or *BF*_10_ < 1/3 (moderate evidence for *H*_0_) for the main effect of memory load in the cross-type-manipulation ANOVA, which examined whether the two target types of materials share a common storage in WM.

For the prediction analysis, 107 of 206 participants underwent MRI experiments, including high-resolution *T*_1_-weighted, resting-state fMRI and task fMRI scanning [part of task fMRI data were reported elsewhere (*65*)]. In the resting-state fMRI analysis, four participants were excluded because of excessive head motion (maximum displacement >3 mm or 3°, or >15% of time points with framewise displacement >0.5 mm), leaving 103 participants in the final fMRI sample (*M* = 19.5, *SD* = 1.3; 61 females and 42 males). For the ROI analysis, a subgroup of 47 participants (*M* = 19.9, *SD* = 0.8; 29 females and 18 males) among 107 participants was included to perform the event *N*-back task and another 34 participants (*M* = 23.3, *SD* = 2.6; 16 females and 18 males) were recruited to perform the event change detection task independently [for details of *N*-back and change detection task, please refer to the study of Zhou *et al.* (*65*)]. For the color-shape binding change detection task, 31 participants were recruited. Four participants were excluded for chance-level performance, two for incomplete fMRI data, and one for excessive head motion (maximum displacement >3 mm or 3°, or >15% of time points with framewise displacement >0.5 mm), resulting in 24 participants (*M* = 23.56, *SD* = 2.01; 18 females and 6 males) in the final analysis.

All participants had normal color vision and normal or corrected-to-normal visual acuity. They provided written informed consent before the experiments and received payment or course credit for their participation. This study was approved by the ethics committee of Zhejiang University [Ethics Approval of Zhejiang University Psychology (2021) no. 051, (2023) no. 081].

The behavioral experiments (for CFA) lasted ~210 min. To control for potential fatigue effects, we divided the testing into three sessions, which were conducted separately for 3 days. In session 1, participants completed seven questionnaires that were irrelevant to our study and were not reported here. In sessions 2 and 3, they completed a series of WM tasks in a dark lab compartment. Resting-state fMRI data and *N*-back task fMRI data were collected after the participants completed all behavioral tests. An independent group performed an event change detection task in the fMRI scanner [preliminary results have been reported in a previous study (*65*)]. The color-shape binding fMRI task was conducted on a separate group of participants, lasting for 40 min. The three psychophysical experiments, each about 50 min long, were conducted on three other independent samples.

### WM measurements for CFA

The stimuli in the 14 WM tasks were generated using MATLAB Psychophysics Toolbox and presented on a black (0, 0, 0) background. Participants sat in a dark room, 60 cm in front of a 17-inch (43.18 cm) cathode ray tube monitor with a resolution of 1024 by 768.

For storage tasks (event, object, and binding storage), the change detection paradigm was adopted, and the number of memory items was set to two, four, and six (for the location storage task, it was four, six, and eight). There were 26 trials under each memory load and 78 trials in each task. Participants could take a break every 26 trials. An eight-trial practice preceded the formal experiment.

To tap CE, we selected four tasks, including the *N*-back, anti-saccade, Ospan, and Sspan tasks. Both the *N*-back and anti-saccade tasks are well-established measures of CE, encompassing updating and inhibition (*68*, *69*). Specifically, the *N*-back task serves to capture the updating component of WM. When the value of *N* is 2 or greater, simply keeping recently presented items in memory is not sufficient to complete the task. Continuous updates to the WM buffer are required to track the current stimulus and facilitate comparisons. In contrast, to complete the anti-saccade task, participants need to inhibit the automatic eye movement toward the cue side of the screen and redirect their gaze in the opposite direction to locate and identify the target. This task taps proponent response inhibition (*70*). The complex span tasks, including Ospan and Sspan, tap both the storage and CE (*4*), and there are studies suggesting that attention control primarily underlies the performance on the complex span tasks (*28*, *71*). The CFA method was used to extract the common variance shared by these four tasks, which mainly reflected CE.

#### Event storage tasks

Event storage tasks included BM and NBM tasks. Following previous studies, we adopted PLDs (*47*) and solid agents (*45*) as two types of BM stimuli and rectangular and circular movements (*33*, *49*) as two types of NBM stimuli.

##### PLD BM

For each PLD movement, 13 light points were placed at the distinct joints of a moving human body to form a coherent and meaningful movement. We selected nine movements from the database of Vanrie and Verfaillie (*72*): cycling, jumping, painting, spading, walking, waving, chopping, paddling, and saluting (see fig. S4B). Every animation consisted of 30 distinct frames, with each frame displayed twice, leading to a 1-s PLD (refresh rate, 60 Hz). Each stimulus subtended a visual angle of ~1.43° (width) by 3.62° (height). The spatial locations of the PLDs were randomly selected from eight evenly distributed spots on an invisible circle with a radius of 4.88° from the screen center.

The procedure of a single trial is shown in fig. S4A. Each trial began with the word “Coca-Cola” (in Chinese) displayed for 500 ms, prompting participants to rehearse it aloud during the whole task. This was to prevent the participants from verbally coding the BM. After an interval of 150 to 350 ms, the memory array appeared for 4 s. Participants were required to remember these stimuli. After a 900-ms retention interval, a red BM was presented at the screen center. Participants were required to determine within 3 s whether the red BM had appeared in the memory array. They pressed the “J” key if it had appeared in the memory array and pressed the “F” key if it had not. The probed red BM remained the same in 50% of the trials and altered to a different action in the remaining 50% of the trials. After the response, there was an interval of 500 to 700 ms between trials.

##### SolidBM

Solid agents were used as the other type of BM stimulus. In this task, BMs were presented in the form of solid agents, which were created using Poser software. We selected nine movements: arm-moving, stretching, jumping, squatting, walking, waving, saluting, turning, and bowing (see fig. S4C). The other settings were the same as those in the PLD BM task.

##### Rectangular movement (RecMove)

The movements of the rectangles were created using a 12-dotted PLD (*33*). In line with the PLD BM stimuli, the dotted rectangle showed nine distinct movements (see fig. S4D). (i) The left and right sides moved downward by 60° relative to their vertical positions and then returned. (ii) The left side moved upward by 45° relative to its vertical position and then returned, while the right half moved upward by 45° relative to its vertical position and then returned. (iii) The left and right halves moved downward by 90° relative to their vertical positions and then returned, while the top side rotated around the middle dot once. (iv) The top side moved upward by 90° relative to its horizontal position and then returned. (v) The top half of the rectangle rotated 90° clockwise relative to its vertical position, while the left bottom half moved upward by 90°. (vi) The top side rotated around the top dot on the right side by 180°, while both the left and bottom sides moved downward by 90°. (vii) The right side moved downward by 45° and then returned. (viii) The left side rotated 180° clockwise around the middle dot, while the right side moved downward by 90° relative to its vertical position. (ix) The top side moved downward by 90° relative to its horizontal position and then returned, while the right side moved 180° clockwise relative to its horizontal position and then returned. The other settings were the same as those in the PLD BM task.

##### Circular movement (CirMove)

We used 13-dotted PLDs of circular movement, containing nine distinct movements (*47*): moving up, moving down, rolling right, rolling left, shrinking, inflating, moving diagonally, splitting horizontally, and splitting vertically (see fig. S4E). The initial radius of each circle is ~1.15°. Each movement lasted for 1 s. The other settings were the same as those in the PLD BM task.

#### Object storage tasks

##### Color

For distinct colors (fig. S4F), we used white (255, 255, 255, in RGB value), yellow (255, 255, 0), lime (0, 255, 0), gray (128, 128, 128), light pink (255, 178, 193), aqua (0, 255, 255), blue (0, 0, 255), red (255, 0, 0), and magenta (255, 0, 255). The spatial locations of the colored squares (1.1° by 1.1°) were distributed at eight spots on an invisible circle with a radius of 4° from the screen center. After a 300- to 400-ms interval, the memory array was displayed for 200 ms. The other settings were the same as those in the PLD BM task.

##### Shape

We replaced the nine colored squares with nine distinct shapes (see fig. S4G; 1.6° by 1.6°). The other aspects were the same as those of the color WM task.

##### Location

Participants were required to remember the locations of four, six, or eight white squares (0.2° by 0.2°) to avoid the ceiling effect. They judged whether the probe (red square) appeared at one of the memorized locations. The stimuli appeared inside an invisible circle with a radius of 5° from the screen center. These squares were evenly distributed in the four quadrants and did not overlap. A fixation (“+”) remained at the screen center from the blank interval after the Coca-Cola presentation until the end of the detection phase. The other settings were the same as those in the color task.

#### Binding tasks

Binding tasks were designed to assess WM capacity when two different features were presented within the visuospatial domains (color-location binding) or across the verbal and visual WM domains (color-letter binding). Participants had to hold them together in WM to respond correctly to the task. The probe could be one of the old items in the memory array in 50% of trials. In the remaining trials, the probe was formed by combining the different dimensions of the two items in the memory array.

##### Color-location binding

Participants were required to remember the bindings between the color and location of the 0.8°-by-0.8° squares. The same colors were chosen from the color task, except that light pink was replaced with green (0, 128, 0). The squares appeared for 200 ms in an area of 10° by 10° in the screen center and were nonoverlapping. Subsequently, a probe appeared at a certain location on the screen, and participants were required to report whether its color matched the memory item at the corresponding location. The fixation settings were the same as those used for the location task.

##### Color-letter binding

Participants were required to remember the bindings between color and capital letter (A, B, C, D, E, H, M, J, and K). Each letter subtended a visual angle of ~1.3° by 1.3° and was presented on an invisible circle with a radius of 4° from the screen center for 200 ms. A colored capital letter then appeared at the screen center. Participants were asked to determine whether any memory item matching both letter and color appeared. No fixations were demonstrated during the task.

#### CE tasks

##### Anti-saccade (Anti)

This task was adapted from the task used by Unsworth and Spillers (*73*). Each trial started with a fixation point displayed for 200, 600, 1000, 1400, or 1800 ms. A cue was then displayed either to the left or right of the fixation point for 100 ms, followed by a 50-ms interval. This procedure was repeated a second time. After the disappearance of the cue, a target letter (B, P, or R) was presented on the opposite side of the second cue for 100 ms, succeeded by a masking stimulus (an H for 50 ms and then an “8” that remained on screen until a response). Participants were required to identify the target letter (B, P, or R) by pressing a corresponding key (1, 2, or 3). There were 15 practice and 40 formal trials. The proportion of correct responses was then recorded.

##### N*-back*

The task began with a 1000-ms fixation. A sequence of black squares then appeared in the eight outer squares of a three-by-three grid (excluding the middle one). Participants had to decide whether each square’s location matched the one showing three items (3-back) before. Each square was presented for 500 ms, with interstimulus fixations of 2000 ms. Participants pressed “F” for a match and “J” otherwise. The task included two 40-trial blocks, preceded by eight practice trials. The proportion of correct responses was then recorded.

#### Other WM tasks

##### Operation span (Ospan)

Participants remembered a set of consonants (F, H, J, K, L, N, P, Q, R, S, T, and Y) while solving arithmetic problems. Three practice sessions preceded the formal trials to familiarize participants with the procedure. In the first session, a simple letter-span task required participants to recall presented consonants in the same order. Each letter was presented in the screen center for 1000 ms. During recall, a 4- by 3-letter matrix was displayed, and participants had to select the letters in the correct order by clicking the box beside the letter. In the second session, the participants were asked to solve 15 arithmetic problems [e.g., (3 × 2) + 6 = ?]. The processing time for each problem was recorded, and the program calculated the mean and SD of the time each participant took after completion. The third session combined the simple span and arithmetic tasks. A mathematical operation was presented first, followed by a letter. The time limit for each operation was fixed to the average time plus 2.5 SD obtained in the second session so that participants had little time to rehearse the letters. After all three practice sessions, the program proceeded to the formal trials, which were similar to the trials in the third practice session. The list length varied randomly from four to eight letters. Three trials were performed for each set size, resulting in a total of 15 trials. The dependent variable was the number of correct lists that recalled all letters in the correct order. Participants repeated the task if the overall accuracy of the math portion was lower than 80%.

##### Symmetry span (Sspan)

Participants had to remember the location of sequences of red squares (1.1° by 1.1°) presented within a 3-by-3 matrix while performing a symmetry judgment task. In line with Ospan, the task started with three practice sessions: two trials of the simple square-span task, 15 symmetry judgment tasks, and three trials of the combined task. In the formal trials, participants first saw a black-and-white 8-by-8 grid pattern and decided whether it was symmetrical along the vertical axis. The time limit was calculated in the same manner as for Ospan. Then, participants were presented with a 4-by-4 matrix with one of the cells filled in red for 650 ms. During recall, a blank 4-by-4 matrix appeared, and participants had to recall the red squares in the same order as they were presented by clicking on the corresponding locations. There were three trials for each set size with a list length ranging from three to six. The same scoring procedure was used as with the Ospan task.

##### Multiple-object tracking (MOT)

Twelve white disks with a viewing angle of 0.25° were presented in an area of 8.5° by 8.5° at the screen center. Disk movement had three stages: marking, tracking, and detection. The marking phase lasted for 3000 ms, during which all disks remained still. However, two, four, or six disks turned red and solid for 2500 ms, prompting the participants to track the position of these disks. Subsequently, all disks became hollow and appeared for 500 ms. The tracking phase lasted for 4500 ms. All discs moved in random directions at speeds ranging from −0.00058°/ms to 0.0064°/ms, and they did not overlap during the movement. The detection phase lasted for a maximum of 1500 ms. All discs stopped moving, and one of the discs turned solid green. Participants were required to determine whether the disc was one of several discs marked in the marking phase. There were 26 trials under each tracking load and 78 trials in total. This task did not require verbal suppression, and the other aspects were the same as those in the PLD BM task.

### Latent variable modeling

For WM storage tasks, the WM capacity for each type of stimulus was estimated using Cowan’s formula (*30*): *K* = *S* × (*H* − *F*), where *K* is the WM capacity, *S* is the number of to-be-memorized stimuli, *H* is the hit rate, and *F* is the false alarm rate. We calculated *K* for each set size for each participant and considered *K*_max_ among the three load conditions as one’s WM capacity (*47*, *49*). For all tasks, univariate outliers were defined as individual scores exceeding 3 SDs from the respective grand mean. Of 2884 observations, 5 met this criterion and were replaced with corresponding cutoff values (*M* ± 3 SD). For the MOT task, we used the formula of Scholl *et al.* (*31*) to derive the effective number of objects tracked: *K* = *S* × (2*P* − 1), where *K* is the effective number of objects tracked, *S* is the number of targets, and *P* is the tracked accuracy under a tracked-load condition.

All structural equation models were estimated using Mplus 8 (www.statmodel.com/). The robust maximum likelihood estimation method that has been developed for nonnormal data was used in modeling estimation (*74*). The evaluation of the fit statistics was based on the criteria recommended by Kline (*75*) and DiStefano (*76*). Specifically, the fit of a model was considered good (or acceptable) if normed χ^2^ (χ^2^/*df*) ≤ 2 (3), root mean square error of approximation (RMSEA) ≤0.05 (0.08), standardized root mean square residual (SRMR) ≤0.05 (0.10), and CFI ≥ 0.95 (0.90). We made model comparisons by considering changes in CFI and AIC. A difference of 0.01 or larger in CFI was considered a substantial difference (*77*). AIC was used to compare nonnested models, in which a model with a smaller AIC was preferred (*76*). The chi-square (χ^2^) difference test was used to compare nested models (*74*).

### Psychophysical experiments

#### Apparatus and stimuli

The experiments were implemented using MATLAB and Psychophysics Toolbox. Stimuli were presented against a gray (128, 128, 128, RGB) background on a 17-inch (43.18 cm) cathode ray tube monitor with a resolution of 1024 by 768 pixels at a 60-Hz refresh rate. Participants were seated in a dark room ~60 cm from the screen. The fixation point subtended a 0.27°-by-0.27° visual angle. Both the memory items and probe items were presented at the screen center.

##### BM

The first seven movements from the PLD BM task were selected. Each action consisted of 15 frames, with each frame displayed twice in succession, leading to a 500-ms duration. In experiment 1, we connected the dots with white line segments to build line-formed BM, where the line segments represent the structure of the human skeleton. The remaining settings remained consistent with those used in the previously mentioned PLD BM task for CFA.

##### RecMove

The first seven movements from the RecMove task were selected. Each comprised 15 frames and was presented for 500 ms. The other settings remained consistent with those in the RecMove task for CFA.

##### Human posture

Seven static body postures were created through Poser software: waving, saluting, pointing, squatting, rejecting, bowing, and stretching (see fig. S6). Each had an approximate size of 2.5° (width) by 4.0° (height). Four of these postures were chosen from the actions in the preceding solid BM task, while the remaining three were additional postures. This selection ensures that each posture has a clear meaning and is distinguishable from each other.

##### Transparent motion

The task in experiments 2 and 3 was adapted from the task used by Valdes-Sosa *et al.* (*78*). The moving dots consisted of 25 red (255, 0, 0) target dots and 10 white (255, 255, 255) distractor dots. The target dots moved together at 4.2°/s in one of seven fixed directions (0°, 51.4°, 102.9°, 154.3°, 205.7°, 257.1°, or 308.6°), while the distractor dots moved together at the same velocity in a different direction from the seven (38.6°, 90.0°, 141.4°, 192.9°, 244.3°, 295.7°, or 347.1°). All dots were randomly generated within a 4.8°-radius circular area at the visual field center. Once a dot moved beyond the circular area, it reappeared at the corresponding position on the opposite side of the circular area. Participants had to retain the moving direction of target dots. Probe items contained only target dots. In experiment 4 of the velocity of transparent motion task, we used 100% motion saliency memory array to reduce the task difficulty. The moving dots consisted of 35 black (0, 0, 0) target dots, whose velocity was set to 0.3°, 0.6°, 1.2°, 2.4°, 4.8°, 9.6°, or 19.2°/s. In each trial, all dots moved in the same direction, which was randomly selected from the seven directions (0°, 51.4°, 102.9°, 154.3°, 205.7°, 257.1°, or 308.6°).

##### Colorized BM

To strictly control the visual attributes, we used colorized BMs as stimuli in experiment 6. We used the same nine PLD BMs as in the event storage task. For distinct colors, we selected brown (124, 77, 37), yellow (255, 255, 0), lime (0, 255, 0), orange (255, 127, 0), pink (255, 178, 193), aqua (0, 255, 255), blue (0, 0, 255), red (255, 0, 0), and magenta (255, 0, 255).

#### Design and procedure

Participants had to maintain stimuli A and B simultaneously in WM (e.g., in experiment 1, A represents BM and B represents RecMove; for experiment 6, A represents BM and B represents the color of BM). Experiments 1 to 5 consisted of five load conditions (2A, 2B, 2A + 2B, 2A + 4B, and 4A + 2B), and experiment 6 contained three conditions (2A + 2B, 2A + 4B, and 4A + 2B), with numbers denoting item counts. Conditions 2A and 2B served the purpose of adding lower WM load conditions and ensuring good WM performance when holding two stimuli. These actions collectively diminish the overall memory task difficulty, boost participants’ confidence in handling higher WM loads (retaining four or six stimuli), and hence avert the emergence of floor effects under such demanding conditions. Each condition included 48 trials, resulting in 240 trials for experiments 1 to 5 and 144 trials for experiment 6.

In Experiments 1 to 5, each trial (see Fig. 3A) began with a 500-ms fixation point in the screen center, followed by a 200-ms blank screen. Then, stimuli were sequentially presented in a random order at the screen center. Each memory item was presented for 500 ms, with a 500-ms interstimulus interval between items. The random presentation ensured that the order of memory item presentation was unpredictable, preventing the formation of chunks in WM. After all memory items, a blank screen of 1500 ms was displayed. Then, a probe item marked with a white frame was presented for 3000 ms. Participants were required to indicate whether this probe had appeared in the memory array by pressing “J” for yes or “F” for no. Except for the conditions where only A or B was present, the detection probability of A and B was 50% each, and the probe had a 50% chance of being in the array. The interval between the two trials varied between 1500 and 2000 ms. Throughout the entire experiment, participants were asked to continuously repeat the phrase “Coca-Cola” to prevent verbal encoding of the memory stimuli.

In experiment 6, we presented four colorized BMs simultaneously for 3 s and asked the participants to memorize both colors and BMs. Both the presented BMs and colors can be either four different ones or two sets of identical pairs, leading to three memory load conditions: two colors and two BMs, two colors and four BMs, and four colors and two BMs. The probe item is either a black BM or a color square in the screen center, which was detected with equal probability.

Participants were given a minimum of 20 practice trials. If they achieved an accuracy of 75% or higher, or completed 40 practice trials, they proceeded to the formal experiment. Participants were allowed to take a break every 30 trials. Accuracy instead of reaction time was emphasized.

#### Analysis

First, a 2 (material type: A versus B)–by–2 (load: 2 and 4) repeated-measures ANOVA (STM_ANOVA) was conducted to investigate the effectiveness of manipulating the memory load of certain memory material, and the load effect was the primary interest to check the effectiveness of load manipulation. Second, a 2 (material type: A versus B)–by–2 (load: 2 and 4) repeated-measures ANOVA (CTM_ANOVA) was conducted to examine whether the memory performance of the fixed-load material was modulated by the load variations of the other material. To be specific, we compared the performance of A under 2A + 2B and 2A + 4B conditions, as well as the performance of B under 2A + 2B and 4A + 2B conditions.

### MRI imaging parameters and preprocessing

#### Resting-state and event-related task fMRI

MRI data for the resting-state and *N*-back tasks were acquired on a 3T Siemens Prisma scanner using a 20-channel coil at the Center for Brain Imaging Science and Technology, Zhejiang University. A *T*_2_*-weighted single-shot echo-planar imaging sequence with multiband acceleration (multiband factor, 4) was used.

The participants were instructed to look at a fixation and remain stationary during the resting-state scanning. The acquisition parameters were as follows: repetition time (TR)/echo time (TE), 1000/34 ms; flip angle, 50°; field of view (FOV), 230 mm by 230 mm; matrix, 92 by 92; voxel size, 2.5 mm by 2.5 mm by 2.5 mm; slice number, 52. High-resolution *T*_1_-weighted anatomical images were collected using a *T*_1_-weighted three-dimensional magnetization-prepared rapid gradient echo sequence with the following parameters: TR/TE, 2300/2.32 ms; flip angle, 8°; FOV, 240 mm by 240 mm; matrix, 256 by 256; voxel size, 0.94 mm by 0.94 mm by 0.9 mm; 208 slices in the sagittal panel. The resting-state scan lasted for 8 min, a duration that is widely accepted in neuroscience research, with their reliability and validity well documented in numerous studies (*39*, *79*, *80*).

MRI data for the event change detection task were acquired on two different sites. Please see our previous study for details of collection procedure and preprocessing steps of both the *N*-back and event change detection tasks (*65*).

#### Object-related task fMRI

A color-shape binding change detection task was adopted as an object-related task. During the task, the to-be-memorized colors and shapes were randomly selected from a pool of six distinct values of each type (fig. S13). Each memory array contained two or four color-shape bindings (1.4° by 1.4°), which were constructed by randomly combining distinct values of two dimensions. The positions of the bindings were randomly selected from 45°, 135°, 225°, and 315° clockwise on a virtual circle 1.7° from the screen center. A one-way (memory load: 2-sets versus 4-sets) within-subject design was adopted, and the order of the two loads was counterbalanced across participants. Each load consisted of two runs, and each run had 24 trials, resulting in a total of 96 trials. Each trial began with a black fixation presented at the screen center for 900 ms, followed by the memory array for 100 ms, a blank interval (randomly chosen from 2, 4, 6, and 8 s), and a probe to identify if it was in the memory array (pressing “4” for yes or “1” for no). After pressing the key, the probe did not disappear immediately and remained present until 2 s. If participants did not respond within 2 s, the response for the trial would be recorded as wrong. In half of the trials, the probe was not included in the memory array.

To mitigate the risk of participants depending solely on memorizing partial bindings to accomplish the task, the introduction of bindings took two different approaches during probe-absent trials. Specifically, in two-thirds of these trials, the binding was generated by randomly combining two features from two distinct bindings within the memory array. In the remaining one-third of probe-absent trials, the binding emerged through the replacement of a feature within an existing binding from the memory array with a previously unused feature. The intertrial interval was randomly chosen from 5, 9, 13, and 17 s with equal probability. Each run lasted 456 s, and the whole experiment lasted 40 min. Before the formal experiment, participants completed a practice session (10 trials) outside the scanner to become familiar with the task.

MRI data for the color-shape binding change detection task were acquired on a 3T Siemens Prisma scanner using a 20-channel coil at the Center for Brain Imaging Science and Technology, Zhejiang University. A *T*_2_*-weighted single-shot echo-planar imaging sequence with multiband acceleration (multiband factor, 4) was used. The acquisition parameters were as follows: TR/TE, 1000/34 ms; flip angle, 62°; FOV, 230 mm by 230 mm; matrix, = 92 by 92; voxel size, = 2.5 mm by 2.5 mm by 2.5 mm; slice number, 52. High-resolution *T*_1_-weighted anatomical images were collected using a *T*_1_-weighted three-dimensional magnetization-prepared rapid gradient echo sequence with the following parameters: TR/TE, 2300/2.32 ms; flip angle, 8°; FOV, 240 mm by 240 mm; matrix, 256 × 256; voxel size, 0.94 mm by 0.94 mm by 0.9 mm; 192 slices in the sagittal panel.

#### Preprocessing of resting-state and object-related task fMRI data

Resting-state and color-shape binding task fMRI data were preprocessed using AFNI (https://afni.nimh.nih.gov/), ANTs (http://stnava.github.io/ANTs/), SPM12 (www.fil.ion.ucl.ac.uk/spm/), and DPABI (https://rfmri.org/DPABI). The first five volumes of the resting-state fMRI data were discarded to allow for signal equilibrium, and the remaining volumes were first corrected for slice acquisition timing and then for head motion using rigid-body transformation. Spatial smoothing was conducted with a 5-mm full-width-at-half-maximum Gaussian kernel after spatial normalization. To further control for head motion influence, the six motion parameters and their temporal derivatives, as well as the first five principal components extracted from the white matter and CSF regions, were regressed out from the smoothed data, and last, band pass filtering (0.01 to 0.1 Hz) was applied.

### Brain-behavior prediction analyses

We calculated the scores of all latent variables to investigate the neural correlates underlying different WM components. We first transformed the scores (accuracies for CE tasks and *K*_max_’s for storage tasks) of each WM task into *z*-scores. According to the structure of model 14, we then used the average of the *z*-scores of the corresponding tasks to attain the score of each first-order factor. For example, BM = 0.5 * (*z*PLD BM + *z*Solid BM). The score of the second-order factor is averaged over the scores of the first-order factors; for example, EVENT = 0.5 * (BM + NBM). Thus, we obtained scores for the EVENT, OBJECT, and CE components. SVR models were trained using the leave-one-out cross-validation method to predict the scores of the CE, EVENT, and OBJECT components derived from factor analysis. The prediction procedure was performed using the MVPANI toolbox (https://github.com/pymnn/MVPANI), which was programmed on the basis of the LIBSVM toolbox (www.csie.ntu.edu.tw/~cjlin/libsvm/).

Feature ranking and selection were performed using a training dataset. For each iteration, one participant was excluded as the testing sample, with the rest forming the training dataset. Within the training dataset, the predictive weight of each edge was determined through SVR model training, and the absolute values of the weights were sorted in descending order. As we used whole-brain connectivity matrices as input features and the number of features is huge, we limited the number of the selected features to be around 200 based on previous studies (*39*, *40*). Thus, five subsets of features ranking from the top 1 to 9‰, with an increase step of 2‰, were used to predict the WM score of the testing sample in the current study (with feature numbers ranging from 35 to 322, more subsets were also tested; see the Supplementary Materials). The correlation coefficient between the predicted WM score and the actual score was calculated to quantify the prediction accuracy with permutation to test the significance of this correlation. For each permutation, the WM scores were shuffled and the same leave-one-out cross-validation prediction procedure was performed to generate a correlation coefficient between the predicted and shuffled WM scores. After 1000 permutations, the significance *P* value was calculated as the percentage of correlation coefficients exceeding the one calculated without shuffling WM scores. We applied FDR correction to account for multiple comparisons relevant to each research question, and the number of comparisons corrected for is explicitly stated in the figure captions.

### ROI analyses of event WM tasks

Both the *N*-back and event change detection event tasks were used to investigate whether the brain regions identified as important by the prediction models participated in WM processing. Both tasks used PLD BM and NBM as stimuli and consisted of two WM loads (0-back and 2-back for the *N*-back task and 2-sets and 4-sets for the event change detection task). For the *N*-back task, BM 0-back, BM 2-back, NBM 0-back, and NBM 2-back were regarded as separate regressors in the GLM analyses. For the change detection task, BM 2-sets, BM 4-sets, NBM 2-sets, and NBM 4-sets regressors were constructed for both encoding and delay periods [for task and GLM details, please refer to the study of Zhou *et al.* (*65*)]. β values were extracted from each load for each stimulus type of the event WM task.

The color-shape binding task was adopted to further confirm the specific role of the left cerebellar node in event cache. For each memory load condition, six regressors were defined: encoding (0.1 s of the memory array), delay (2/4/6/8 s of the delay after the memory array), response (2 s) for trials that were correctly responded, encoding, delay, and response for trials with wrong response.

We conducted two-sided one-sample *t* tests to test the significance of activation and two-sided paired *t* tests to compare activation differences between low and high loads for each stimulus type (BM and NBM, as well as the binding task), and the effect size (Cohen’s *d*) was also estimated by using JASP 0.17.2.1 (https://jasp-stats.org/).

In addition, we conducted time-series analyses for the event change detection task to investigate the BOLD signal change percentage during the task. Specifically, for each participant, we extracted the average time course of the ROI and segmented it according to the onset time of each encoding period. We then averaged the time-course segments of each load for each stimulus type. The resultant time courses were further converted to the percent signal change for each condition by subtracting the value of the corresponding time points of the baseline trial and then dividing by the baseline trial value. We observed two peaks in the time series, which corresponded to the encoding and probe periods. Accordingly, we selected signals between 13 and 15 s for the delay phase (*50*). Last, we conducted two-sided paired *t* tests to test the load effects (4-sets > 2-sets) for each stimulus type.

The correlation between brain activation and behavioral performance on the WM storage tasks was also examined. To reduce the number of multiple comparisons, we calculated a combined behavioral index for WM performance and a combined index for brain response in the change detection tasks (including the event task and color-shape binding task). We first did *z*-transform for the accuracy and brain activation for each WM load of each stimulus type. Then, we calculated the average of *z*-transformed accuracies for two WM loads of each stimulus type as WM performance, as well as the average of *z*-transformed activation for two WM loads of each stimulus type as brain activation. The Pearson’s correlation efficient was calculated between brain activation and WM performance for each stimulus type. Given that information maintenance is the key feature in WM, we only considered the delay period when performing statistical comparisons.
