# Supplementary material for: Event cache: An independent component in working memory
Source: Sci Adv. 2025 May 23;11(21):eadt3063. doi: 10.1126/sciadv.adt3063 (PMC12101497; doi:10.1126/sciadv.adt3063)
Supplement: Supplementary file 1 — Supplementary Text Figs. S1 to S16 Legends for tables S1 to S10 [file sciadv.adt3063_sm.pdf]

Science Advances
AAAS

Supplementary Materials for

Event cache: An independent component in working memory

Hui Zhou *et al.*

Corresponding author: Mowei Shen, mwshen@zju.edu.cn; Tengfei Wang, tfwang@zju.edu.cn;
Yuzheng Hu, huyuzheng@zju.edu.cn; Zaifeng Gao, zaifengg@zju.edu.cn

Sci. Adv. **11**, eadt3063 (2025)
DOI: 10.1126/sciadv.adt3063

The PDF file includes:

Supplementary Text
Figs. S1 to S16
Legends for tables S1 to S10

Other Supplementary Material for this manuscript includes the following:

Tables S1 to S10

Supplementary Text

1. Extended technical descriptions of results for Eye-tracking experiment

1.1 The purpose to conduct eye-tracking experiments

The eye-tracking experiment aimed to determine whether participants encoded the event stimuli in the simultaneous-display change detection paradigm as discrete items or as a single complex entity. We used biological movement (BM) as the representative stimulus. The experimental setup was similar to the PLDBM task in the confirmatory factor analysis (CFA) section, with differences in set size and spatial configuration of the memory array. The set size was fixed at four, with items presented at four adjacent locations out of eight possible spots. This configuration was chosen because the Kmax values in the CFA analyses primarily involved set sizes of 4 and 6, and participants likely processed the items as a whole in this context. Additionally, items were presented at adjacent locations to minimize sequential saccades. If participants still made sequential saccades between the four BM items presented simultaneously at adjacent locations, it would suggest they processed each stimulus as a separate item (or event), which can be extended to the set sizes of 4 and 6 tested in the CFA experiments.

1.2 Methods

Twelve participants were recruited from Zhejiang University (11 females and 1 male between the ages of 19 and 34; M = 23.8, SD = 5.3). They all provided signed informed consent, had normal color vision and normal or corrected-to-normal visual acuity, and received payment for their participation.

The spatial locations of the PLDs were randomly selected from eight evenly distributed spots on an invisible circle with a radius of 4° from the screen center. The memory array consisted of four BM stimuli, randomly presented either on the left or right side of the circle with a 50% probability. The experiment began with a 13-point calibration of the eye tracker, followed by a validation stage. Recalibration was performed if the participant's head position shifted, or if the initial fixation point deviated from the screen center by more than 1° of visual angle before the trial began. The main experiment consisted of 60 trials, divided into four blocks. The rest of the procedure was identical to the PLDBM task (see fig. S1a).

Fixations were recorded using an Eyelink Portable Duo eye tracker system (sampling rate 1000 Hz). Only correctly responded trials were included in the analysis. The analysis focused on the 4000 ms epoch during which the stimuli were presented. The regions of interest (ROIs) were defined as the eight square areas with a visual angle of 2.62° (120 pixels) where the stimuli appeared. We calculated the total fixation duration for each stimulus presentation location under left and right conditions within the epoch of interest. Fixation trajectories were also plotted for all participants.

1.3 Extended Results

The overall accuracy was 87%. fig. S1b illustrates the fixation trajectories for two representative trials. In each trial, participants sequentially fixated on different stimulus locations through rapid saccades. For example, in the trial shown on the left, the participant's fixations were in the order of Fix1 (389 ms), Fix2 (919 ms), Fix3 (1380 ms), and Fix4 (1101 ms). In the trial on the right, the sequence was Fix1 (103 ms), Fix2 (726 ms), Fix3 (848 ms), Fix4 (1072 ms), and Fix5 (1429 ms), with Fix1 and Fix3 targeting at the same item. These trajectories indicate that participants rapidly shifted their attention between stimuli. fig. S1c displays the fixation and saccade trajectories for all 12 participants. Each subplot represents a participant, with dark dots indicating fixation points and light dashed lines representing saccades. The trajectories reveal that participants' fixations were concentrated on the stimulus locations, suggesting that they focused their attention on the relevant stimuli. The results demonstrate that participants processed each BM stimulus sequentially through rapid saccades and fixations.

fig. S1d shows the average fixation duration for each location under both the left and right conditions. Under the left condition, the fixation durations were 775 ms, 1009 ms, 913 ms, and 778 ms, with minimal fixations (0–3 ms) at non-stimulus locations. Under the right condition, fixation durations were 722 ms, 868 ms, 1022 ms, and 885 ms, with minimal fixations (0–2 ms) at non-stimulus locations. Fixation durations were significantly longer at the stimulus presentation areas compared to other areas, indicating focused attention on the relevant stimuli. The average dwell time of each stimulus was around 1 second, which is consistent with the lifetime of an event in our study. These findings suggest that participants do not treat multiple dynamic BM stimuli as a single integrated event, but rather process them as discrete items (or events).

2. Extended technical descriptions of results for MVPA analysis

The primary goal of the MVPA analysis is to investigate the specific role of cerebellar ROI in event processing, addressing the limitations of univariate analyses. The MVPA analysis has two main objectives: (1) to verify whether the cerebellar ROI specifically decodes event-load information, and (2) to confirm whether it specifically decodes event-content information.

2.1 Methods

2.1.1 MVPA analysis rationale

(1) Event-load classification

To achieve the first objective, our analysis approach was as follows: based on the

first-level GLM modeling results from the N-back, event change detection, and object change detection tasks, we used the activation values of each voxel in the cerebellar ROI from the biological movement (BM) low and high WM load conditions in the N-back task (i.e., BM-0back and BM-2back) as the training set to train a classifier for event load classification. This classifier was then applied to predict the NBM (non-biological movement) low and high load conditions in the event change detection task (i.e., NBM 2sets and NBM 4sets), as well as color-shape binding (CSB) low and high load conditions in the object change detection task (i.e., CSB 2sets and CSB 4sets, fig. S14A). If the cerebellar ROI specifically encodes event-related load information, the classifier should accurately distinguish between the NBM 2sets and NBM 4sets in the event change detection task, but should not differentiate between the CSB 2sets and CSB 4sets in the object change detection task. To control for the influence of stimulus materials (BM vs. NBM), we further explored using N-back's NBM-0back and NBM-2back as the training set to predict the BM 2sets and BM 4sets in the event change detection task and the CSB 2sets and CSB 4sets in the object change detection task.

(2) Event-content classification

To validate the second hypothesis, we conducted the investigation in two steps. First, we aimed to verify whether the cerebellar ROI encodes event-content information. We used the activation values of each voxel in the cerebellar ROI under the BM condition in the event change detection task and the CSB condition in the object change detection task to train an event-object category classifier (combining low and high loads). This classifier was then evaluated using leave-one-out cross-validation to determine whether it could distinguish between the two conditions. Next, we applied the classifier to classify the BM and NBM conditions in the N-back task. If the cerebellar ROI encodes event-general content information rather than information specific to different stimulus materials within the event category (BM vs. NBM), the classifier should fail to accurately classify the BM and NBM conditions. Similarly, to control for the influence of stimulus materials, we replaced the BM condition in the training set with the NBM condition and performed the same analysis.

Notably, all activations mentioned in the above analysis regarding the change detection task refer to the activations during encoding or delay phases.

2.1.2 Permutation test for significance determination

To test whether the classifier's accuracy is above chance level, we performed permutation tests to assess the significance of the accuracy. Specifically, we randomly shuffled the labels in the training set, trained the model based on the shuffled labels, and predicted the test set. The classification accuracy was then calculated, and this procedure was repeated 5000 times to obtain 5000 random accuracy values. The proportion of random accuracy values exceeding the true accuracy was calculated and used as the p-value (a p value smaller than 0.05 was considered as significant).

2.2 Extended Results

2.2.1 Extended Results for event-load classification

2.2.1.1 The classifier trained by the BM loads of the N-back task can distinguish the NBM but not the CSB loads in the change detection tasks

As shown in fig. S14B, the MVPA results demonstrated that the classifier was able to accurately distinguish between NBM low load and high load (using activation values during the encoding phase: classification accuracy = 0.677, permutation p = 0.004; using activation values during the delay phase: classification accuracy = 0.735, permutation p = 0.018), but cannot distinguish low load from high load in the object change detection task (encoding phase: classification accuracy = 0.500, permutation p = 0.457; delay phase: classification accuracy = 0.500, permutation p = 0.423, fig. S14B).

2.2.1.2 The classifier trained by the NBM loads of the N-back task can distinguish the BM but not the CSB loads in the change detection tasks.

As shown in fig. S14C, the MVPA results demonstrated that the classifier trained by the NBM task was able to accurately distinguish between BM low load from high load (using activation values during the encoding phase: classification accuracy = 0.735, permutation p = 0.006; using activation values during the delay phase: classification accuracy = 0.677, permutation p = 0.046; fig. S14C), but cannot distinguish low load from high load in the object change detection task (encoding phase: classification accuracy = 0.500, permutation p = 0.446; delay phase: classification accuracy = 0.520, permutation p = 0.280, fig. S14C).

The two MVPA analyses collectively support the CFA and behavioral experimental results, indicating that both BM and NBM reside within the event cache, whereas CSB is stored in a distinct storage cache.

2.2.2 Extended Results for event-content classification

2.2.2.1 The classifier trained by the event and object can distinguish these two task conditions

We first trained a classifier using activation values from the BM condition (event) in the event change detection task, as well as the CSB condition (non-event) in the object change detection task. We performed leave-one-subject-out cross-validation, using the data from all subjects except one for training and the data from the held-out subject for testing (fig. S15A). The classifier was able to accurately distinguish between the event-related BM and non-event-related CSB conditions in both the encoding and delay phases (encoding: classification accuracy = 0.853, permutation $p < 0.001$; delay: classification accuracy = 0.741, permutation $p < 0.001$; fig. S15B).

We also trained a classifier using activation values from the NBM condition (event) in the event change detection task, as well as the CSB condition (non-event) in the object change detection task, and tested whether this classifier could classify these two conditions, the MVPA analysis showed similar results as above (encoding phase: classification accuracy = 0.879, permutation $p < 0.001$; delay phase: classification accuracy = 0.724, permutation $p < 0.001$; fig. S15B).

In short, the cerebellum ROI responds differently to event and object tasks.

2.2.2.2 The classifier trained by the event and object cannot distinguish BM and NBM task conditions

To further assess whether the cerebellum's activation is specific to event-related content (rather than distinguishing between BM and NBM within the event category), we trained the same classifier as above (a. a model trained using activation from BM (event) and CSB (non-event) conditions in the event/object change detection tasks; b. a model trained using activation from NBM (event) and CSB (non-event) conditions in the event/object change detection tasks, fig. S16A) to distinguish between BM and NBM conditions in the N-back task. Critically, the classifier was unable to classify BM and NBM conditions, regardless of whether activation data from the encoding or delay phases were used (minimum permutation $p > 0.479$, fig. S16B, C). The results remain unchanged when exchanging true labels between BM and NBM conditions in the N-back task (minimum permutation $p > 0.505$).

The two MVPA analyses collectively support the CFA and behavioral experimental results, indicating that the representations of BM and NBM belong to the same category, while the representation of CSB is stored in a distinct format.

3. Extended technical descriptions of Experiment S1's results

Six psychophysical experiments further confirmed the independence of event cache in WM. To further verify and extend the results, we conducted an extra experiment comparing two types of object stimuli: transparent motion vs. human posture (fig. S6). Twenty participants were recruited from Zhejiang University for each experiment (13 females, 7 males between the ages of 19 and 33 (M = 23.0, SD = 3.1)). They all provided signed informed consent, had normal color vision and normal or corrected-to-normal visual acuity, and received payment for their participation. Our model predicted that human postures and transparent motion share one common WM buffer (object cache). We tested this prediction by replacing the biological movement in Experiment 3 with transparent motion (memorizing the direction of transparent motion). We predicted a WM capacity competition between the two types of stimuli. The increase of WM load of the stimulus type to be detected significantly decreased the memory accuracy (main effect of Load: F (1, 19) = 24.057, p <0.001, η_p^2 = 0.559, BF_{10} = 159.356, fig. S7), indicating the load manipulation was effective. The main effect of material type [F (1, 19) = 3.316, p = 0.084, η_p^2 = 0.149, BF_{10} = 1.170] and the interaction [F (1, 19) = 2.715, p = 0.116, η_p^2 = 0.125, BF_{10} = 1.017] were not significant.

Meanwhile, the increase of WM load of the stimulus type not to be detected also had a significant load effect on memory accuracy of the stimulus type being detected (main effect of Load: F (1, 19) = 20.703, p < 0.001, η_p^2 = 0.521, BF_{10} = 6.268), suggesting that WM capacity competition occurred and human postures and transparent motion shared a common storage space in WM. The main effect of material type [F (1, 19) = 0.002, p = 0.968, η_p^2 = .8.751e-5, BF_{10} = 0.340] and the interaction [F (1, 19) = 0.821, p = 0.376, η_p^2 = 0.041, BF_{10} = 0.479] were nonsignificant. This result was in line with our prediction that human posture is stored as object in WM.

Fig. S1.

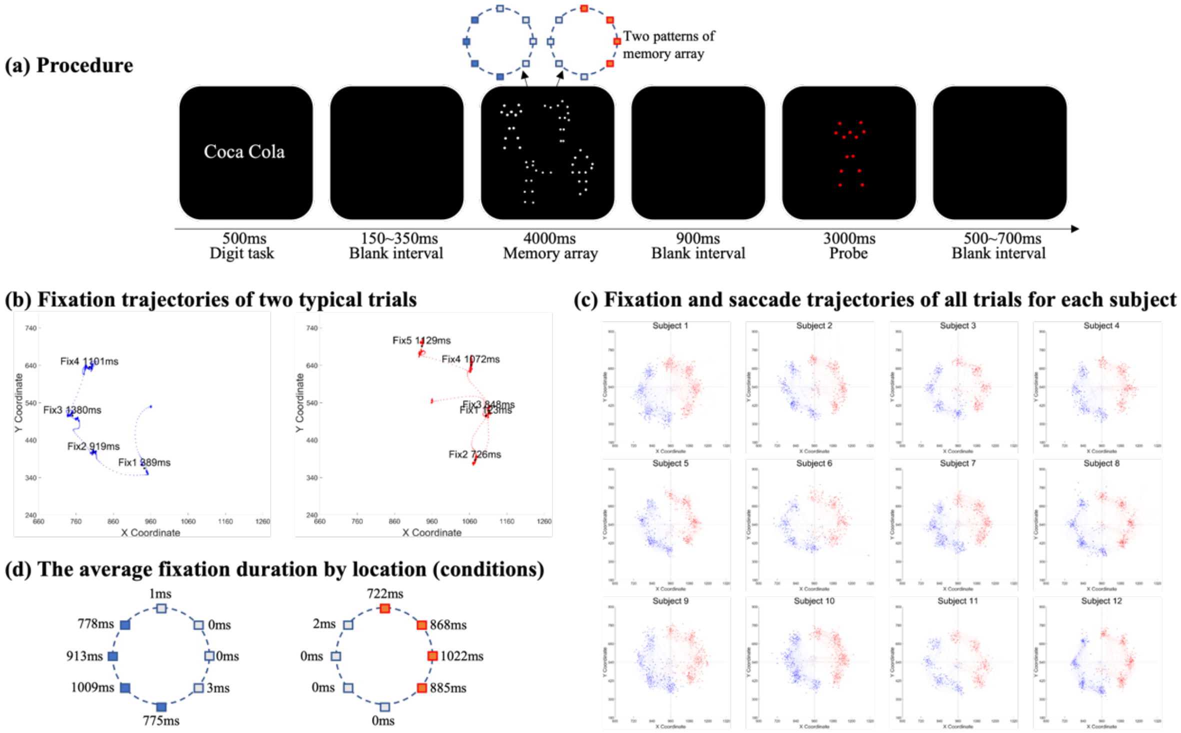

Fig. S1. The eye-tracking experiment. (a) In the memory array, four biological movement (BM) stimuli were presented in four circularly aligned positions, either on the left or right side of a virtual circle. A probe motion was shown to test whether participants retained the information in working memory (WM). Participants were instructed to repeat the word “coca-cola” continuously during the task to prevent verbal encoding strategies. (b) A typical fixation trajectory when the memory array appeared on the left or right side of the circle. (c) Results of all fixation and saccade trajectories across all trials for each participant (n = 12). Blue represents fixations and trajectories when the memory array was on the left side, while red represents those when it was on the right side. These results demonstrate that when multiple dynamic items appear simultaneously, participants use rapid saccades to sample each item sequentially, enabling each stimulus to be processed as an independent event in WM. (d) Average fixation duration by location under different conditions. The numbers adjacent to each square represent the average fixation duration in milliseconds (ms) for the respective position. The results indicate that participants exhibited significantly longer fixation durations at the locations where the BM stimuli were presented, suggesting focused attention on these areas. In contrast, the control positions, where no BM stimuli were present, were associated with minimal fixation durations.

Fig. S2.

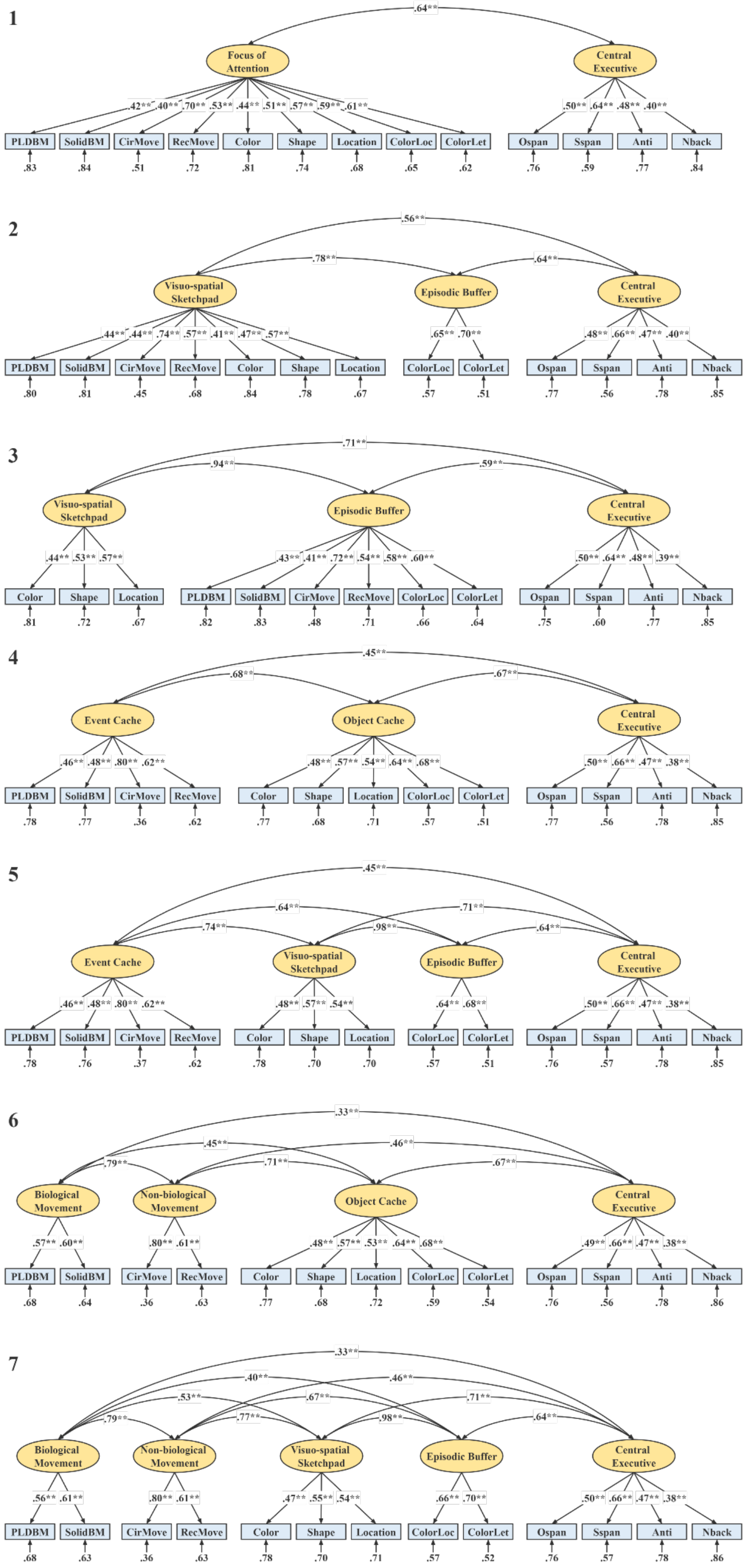

1
Focus of Attention
Central Executive
.64**
.42** .40** .70** .53** .44** .51** .57** .59** .61**
.50** .64** .48** .40**
PLDBM SolidBM CirMove RecMove Color Shape Location ColorLoc ColorLet
Ospan Sspan Anti Nback
.83 .84 .51 .72 .81 .74 .68 .65 .62
.76 .59 .77 .84
2
Visuo-spatial Sketchpad
Episodic Buffer
Central Executive
.56** .78** .64**
.44** .44** .74** .57** .41** .47** .57**
.65** .70**
.48** .66** .47** .40**
PLDBM SolidBM CirMove RecMove Color Shape Location
ColorLoc ColorLet
Ospan Sspan Anti Nback
.80 .81 .45 .68 .84 .78 .67
.57 .51
.77 .56 .78 .85
3
Visuo-spatial Sketchpad
Episodic Buffer
Central Executive
.71** .94** .59**
.44** .53** .57**
.43** .41** .72** .54** .58** .60**
.50** .64** .48** .39**
Color Shape Location
PLDBM SolidBM CirMove RecMove ColorLoc ColorLet
Ospan Sspan Anti Nback
.81 .72 .67
.82 .83 .48 .71 .66 .64
.75 .60 .77 .85
4
Event Cache
Object Cache
Central Executive
.45** .68** .67**
.46** .48** .80** .62**
.48** .57** .54** .64** .68**
.50** .66** .47** .38**
PLDBM SolidBM CirMove RecMove
Color Shape Location ColorLoc ColorLet
Ospan Sspan Anti Nback
.78 .77 .36 .62
.77 .68 .71 .57 .51
.77 .56 .78 .85
5
Event Cache
Visuo-spatial Sketchpad
Episodic Buffer
Central Executive
.45** .64** .71** .74** .98** .64**
.46** .48** .80** .62**
.48** .57** .54**
.64** .68**
.50** .66** .47** .38**
PLDBM SolidBM CirMove RecMove
Color Shape Location
ColorLoc ColorLet
Ospan Sspan Anti Nback
.78 .76 .37 .62
.78 .70 .70
.57 .51
.76 .57 .78 .85
6
Biological Movement
Non-biological Movement
Object Cache
Central Executive
.33** .45** .46** .79** .71** .67**
.57** .60**
.80** .61**
.48** .57** .53** .64** .68**
.49** .66** .47** .38**
PLDBM SolidBM
CirMove RecMove
Color Shape Location ColorLoc ColorLet
Ospan Sspan Anti Nback
.68 .64
.36 .63
.77 .68 .72 .59 .54
.76 .56 .78 .86
7
Biological Movement
Non-biological Movement
Visuo-spatial Sketchpad
Episodic Buffer
Central Executive
.33** .40** .46** .53** .67** .71** .79** .77** .98** .64**
.56** .61**
.80** .61**
.47** .55** .54**
.66** .70**
.50** .66** .47** .38**
PLDBM SolidBM
CirMove RecMove
Color Shape Location
ColorLoc ColorLet
Ospan Sspan Anti Nback
.68 .63
.36 .63
.78 .70 .71
.57 .52
.76 .57 .78 .86

Fig. S2. Latent variable models based on Cowan and Baddeley's models (1–3), models considering an independent event cache in WM (4–5), and models with two types of event cache (6–7). PLDBM = point-light display biological movement; SolidBM = solid biological movement; CirMove = circle movement; RecMove = rectangle movement; ColorLoc = color-location binding; ColorLet = color-letter binding; Ospan = operation span; Sspan = symmetry span; Anti = anti-saccade. Asterisks indicate significant paths or loadings (** p <0.01). In the fig., circles represent latent variables, and squares represent observed indicators. The single-headed arrow points from a latent factor to an observed indicator, and the double-headed arrow indicates the correlation between latent variables. The single-headed arrow on an observed indicator indicates the error term.

Fig. S3.

8

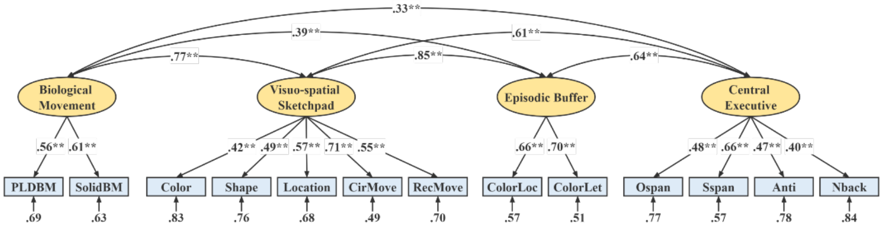

9

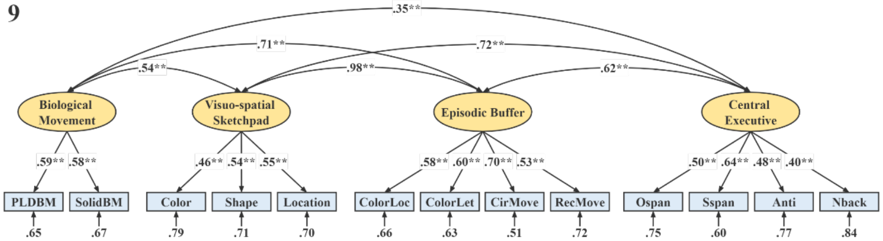

10

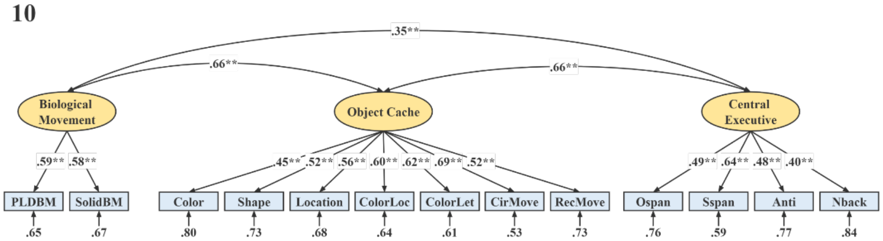

11

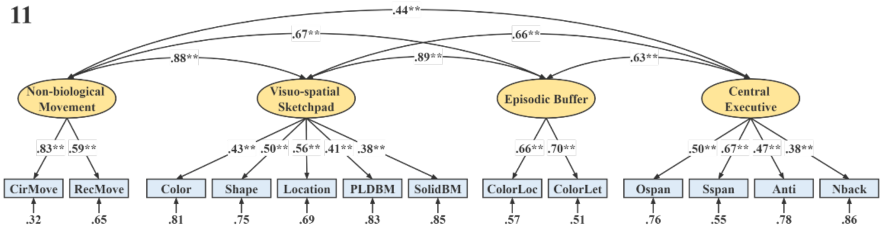

12

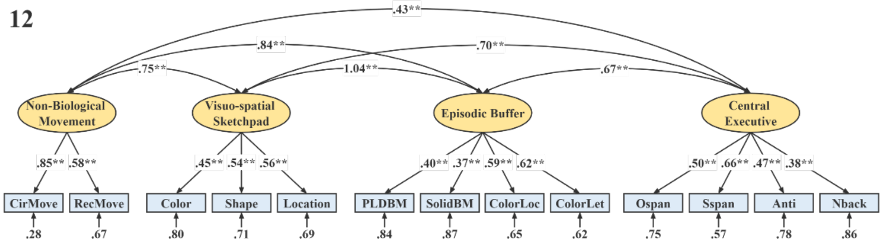

13

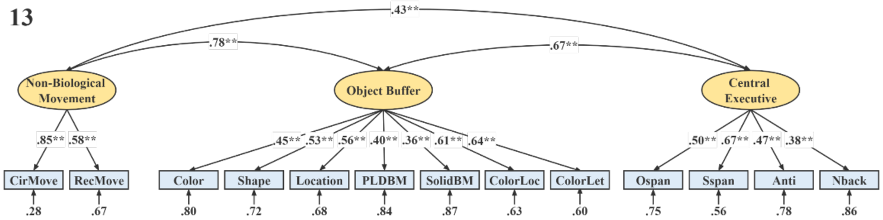

Fig. S3. Latent variable models integrating biological movements and non-biological movements into visuo-spatial sketchpad, episodic buffer and object cache, respectively. We established several alternative models to test whether biological and non-biological movement factors can be integrated into other factors. In **Models 8-10**, the non-biological movement tasks were loaded on the visuo-spatial sketchpad (Model 8), episodic buffer (Model 9), or object cache (Model 10). In **Models 11-13,** the biological movement tasks were loaded on the visuo-spatial sketchpad (Model 11), episodic buffer (Model 12), or object cache (Model 13). The results showed that all six models were unacceptable (table S3). These results further suggest that events, including biological and non-biological movements, are represented separately from objects in the working memory (WM). PLDBM = PLDs biological movement; SolidBM = solid biological movement; CirMove = circle movement; RecMove = rectangle movement; ColorLoc = color-location binding; ColorLet = color-letter binding; Ospan = operation span; Sspan = symmetry span; Anti = anti-saccade. Asterisks indicate significant paths or loadings (** p <.01).

Fig. S4.

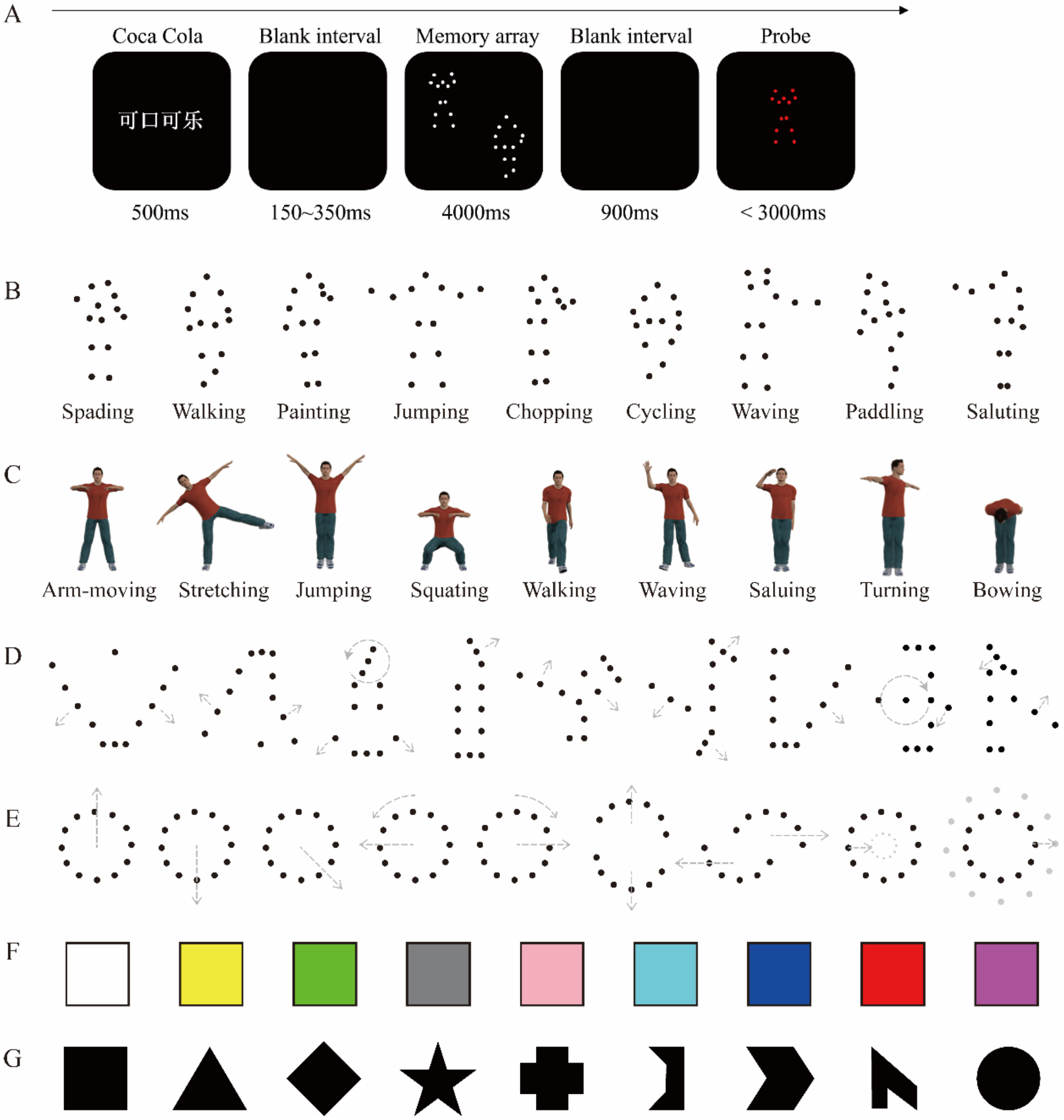

Fig. S4. A schematic illustration of a single trial in PLDs biological movement task (A) and stimuli used in WM tasks (B-G). Example frames for the PLDs biological movement (BM) stimuli (B), solid biological movement (C), movements of rectangles (D), and movement of circles (E). The grey arrow was used to illustrate the moving direction and did not appear in the experiment. Example stimuli of color (F), and shape (G).

Fig. S5.

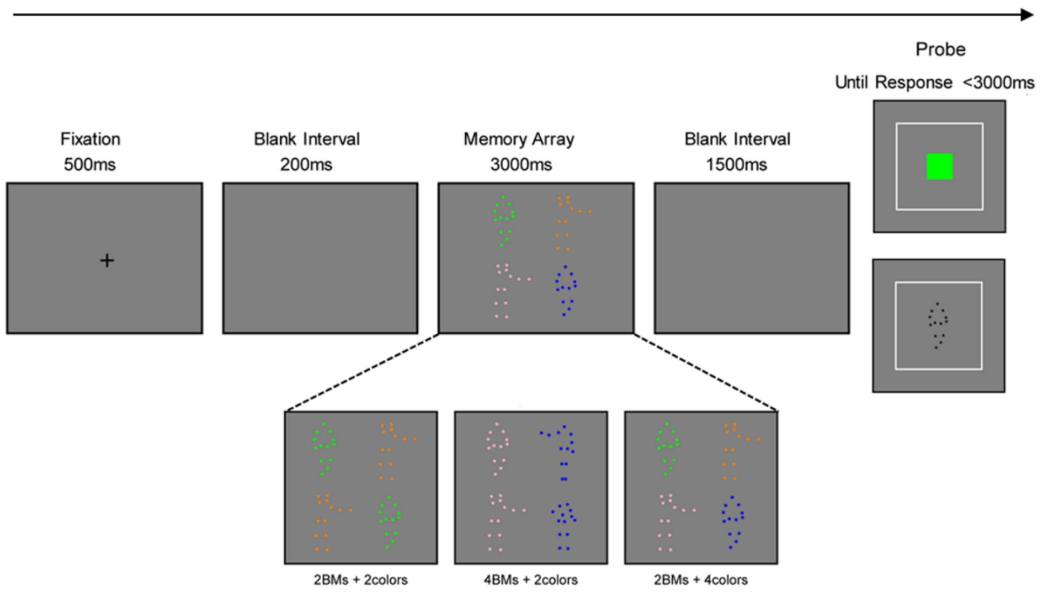

Fig. S5. Design of the dual-task experiment 6: A schematic illustration of a single trial. Each trial began with the presentation of a fixation point at the screen center for 500ms, followed by a 200ms blank screen. After the blank screen, four colorized BMs were presented simultaneously, which may contain two distinct BMs and two distinct colors, two distinct BMs and four distinct colors, or four distinct BMs and two distinct colors. Participants were tasked with retaining both sets of information and determining whether the probed BM or color appeared in the memory array.

Fig. S6.

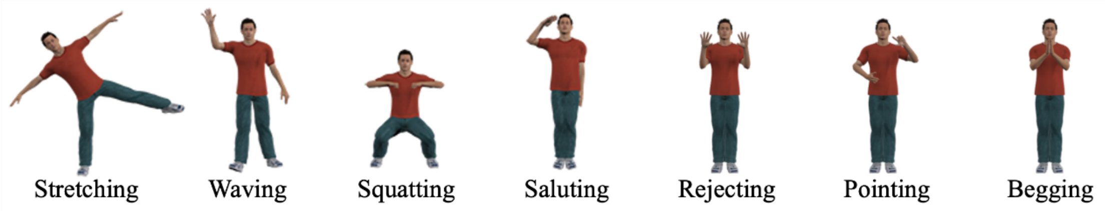

Fig. S6. Memory stimuli of human postures used in dual-task Experiment S1.

Fig. S7.

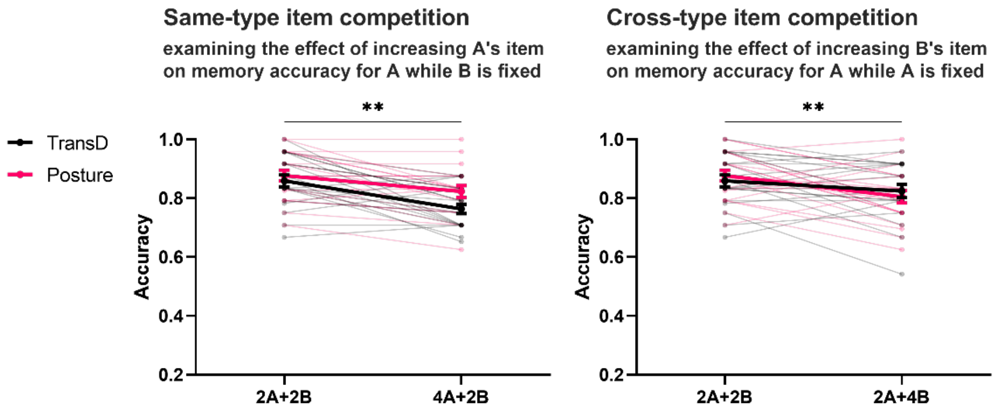

Fig. S7. The memory performance for different types of stimuli in Experiments S1. The left chart shows the results of memory accuracy for Type A stimuli under different loads (2 or 4) of A while keeping the load of B fixed at two, to examine the effectiveness of WM load manipulation. The right chart shows the results of memory accuracy for fixed-load materials (Type A, load 2) under different loads of the other type of materials (Type B, load 2 or 4), to investigate whether the two types of stimuli compete the storage capacity. The legend in the fig. represents the stimulus used as Type A in the x-axis. Note: TransD = direction of transparent motion. Error bar stands for standard error of mean. ** $p < 0.01$.

Fig. S8.

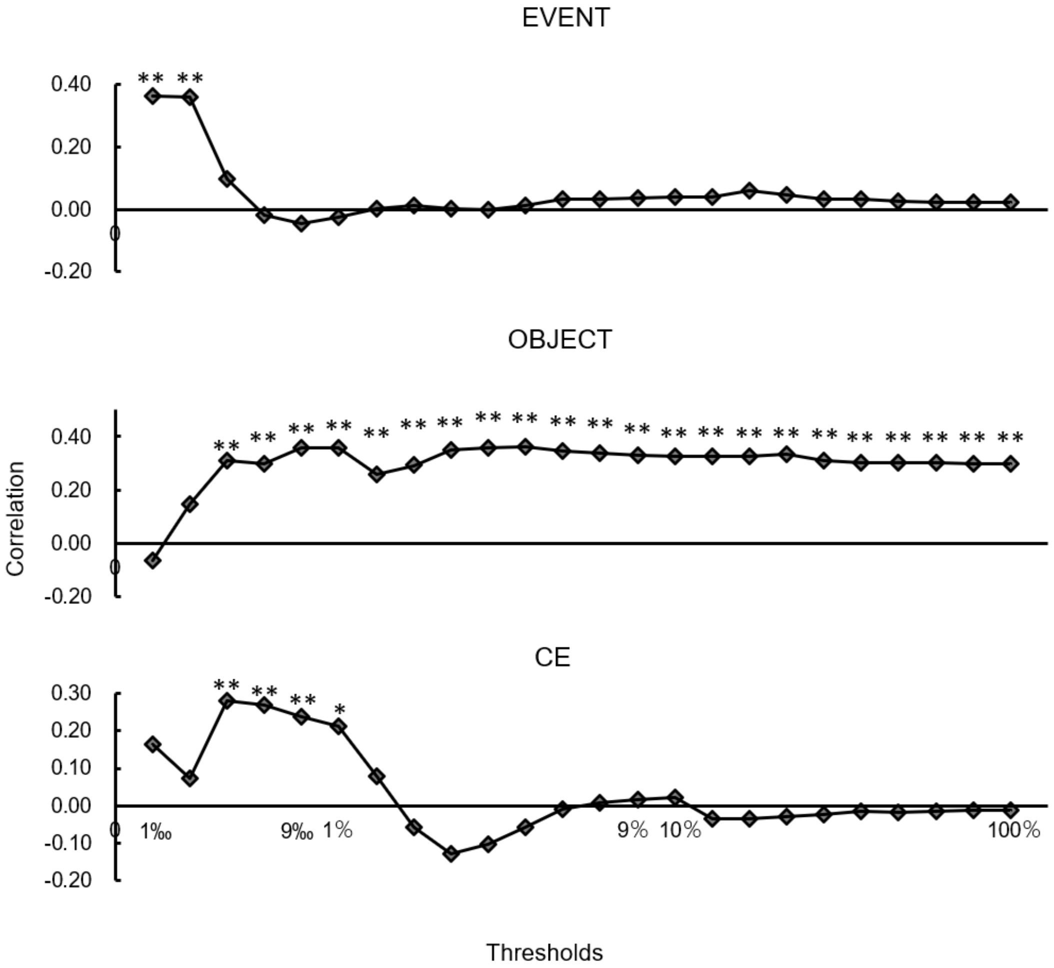

Fig. S8. Prediction performance of three components with more thresholds. Threshold ranges from 1‰~9‰, with a step of 2‰, then from 1%~9%, with a step of 1%, then from 10%~100%, with a step of 10%. *0.01< p <0.05. ** p < 0.01 (without correcting for multiple comparisons). CE = central executive.

Fig. S9.

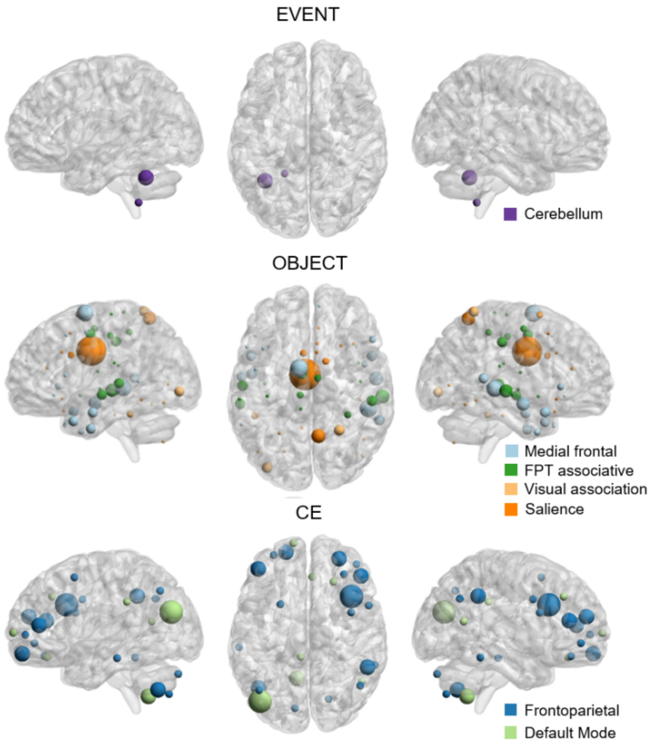

Fig. S9. Node degrees of brain regions within networks important for prediction of the three WM components. The physical size of a node within each chart is proportional to its node degree. FPT associative = frontal-parietal-temporal associative; CE = central executive.

Fig. S10.

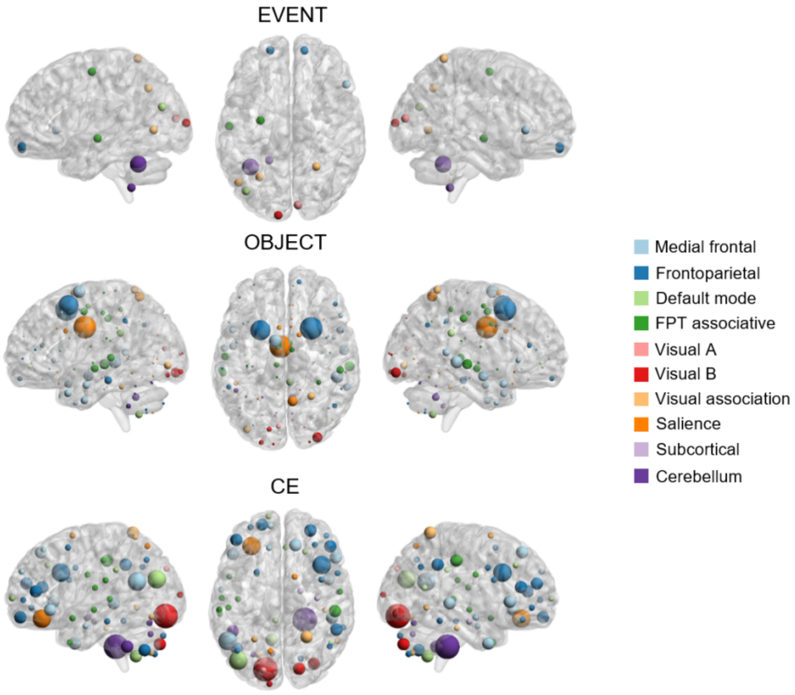

Fig. S10. Node degree illustration for all networks of each WM component. Abbreviations: FPT associative = frontal-parietal-temporal associative; CE = central executive.

Fig. S11.

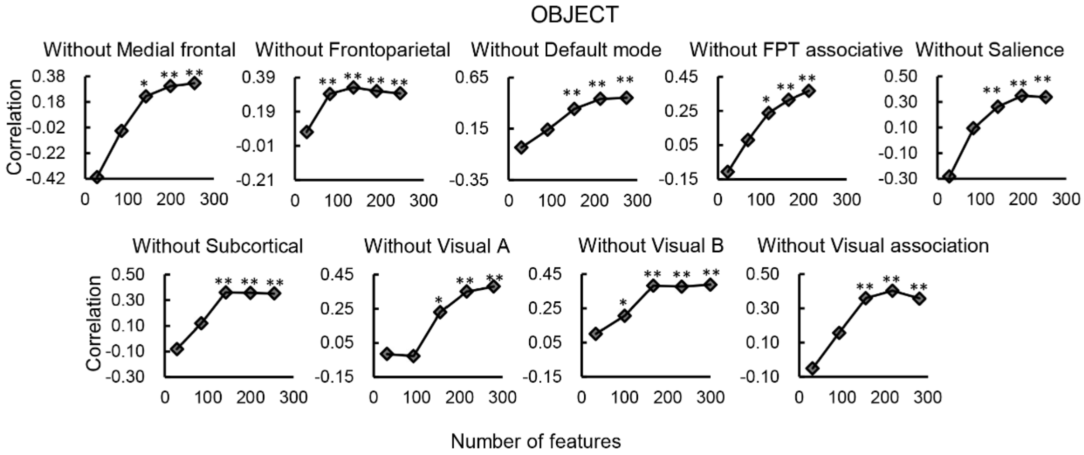

Fig. S11. Prediction performance of OBJECT component after removing one brain network. The OBJECT component could be predicted no matter which network was removed. *0.01< p <0.05. ** $p < 0.01$. FPT associative = frontal-parietal-temporal associative. (without correcting for multiple comparisons)

Fig. S12.

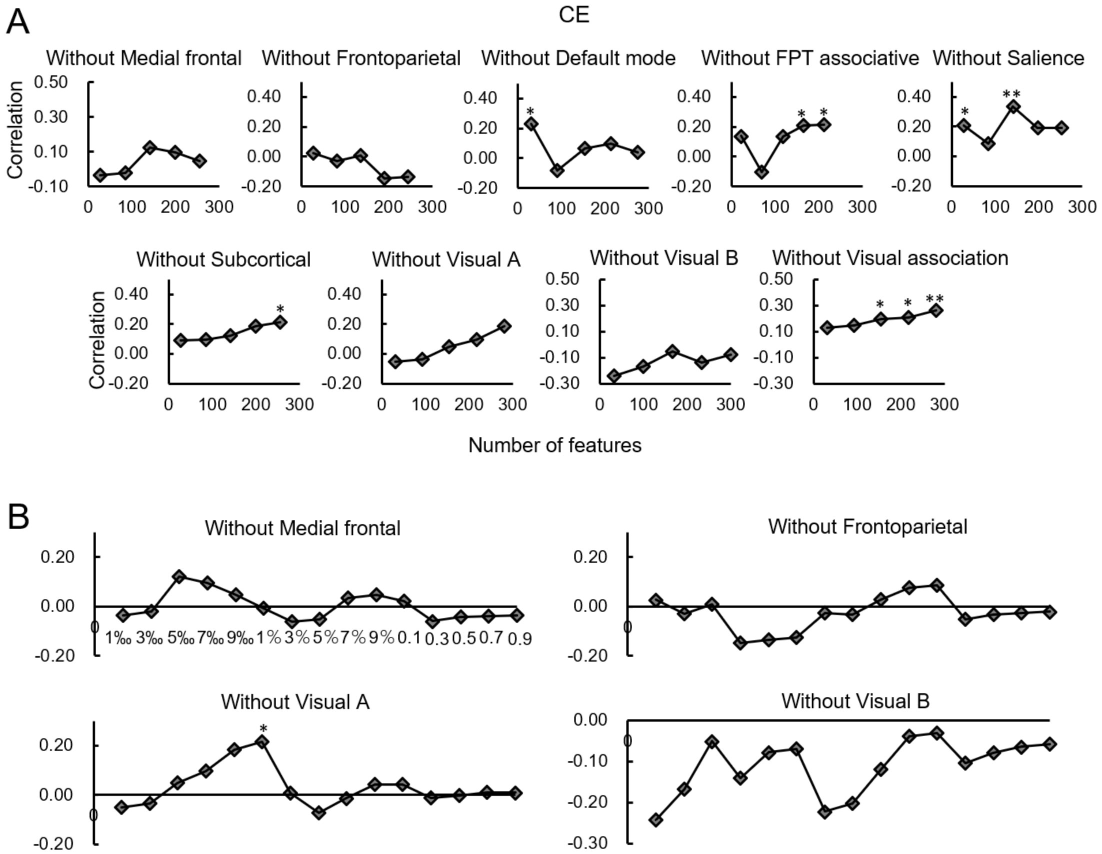

Fig. S12. Prediction performance of CE component after removing one brain network. (A). Prediction performance with a threshold of 1‰~9‰. CE component could not be predicted when the medial frontal, frontoparietal, visual A, or Visual B network was removed. (B). Prediction performance with a threshold of 1‰~90%. Without medial frontal, frontoparietal, or Visual B network, the CE component could not be predicted, whereas under a threshold of 1%, the CE component could still be predicted when the Visual A network was removed. *0.01< p <0.05. ** p < 0.01. FPT associative = frontal-parietal-temporal associative. (without correcting for multiple comparisons)

Fig. S13.

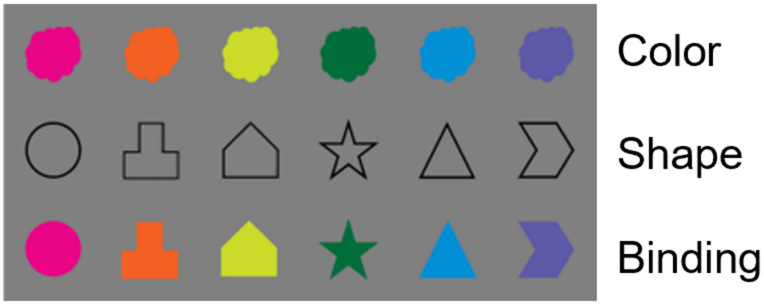

Fig. S13. Examples of stimuli used in the color-shape binding task.

Fig. S14.

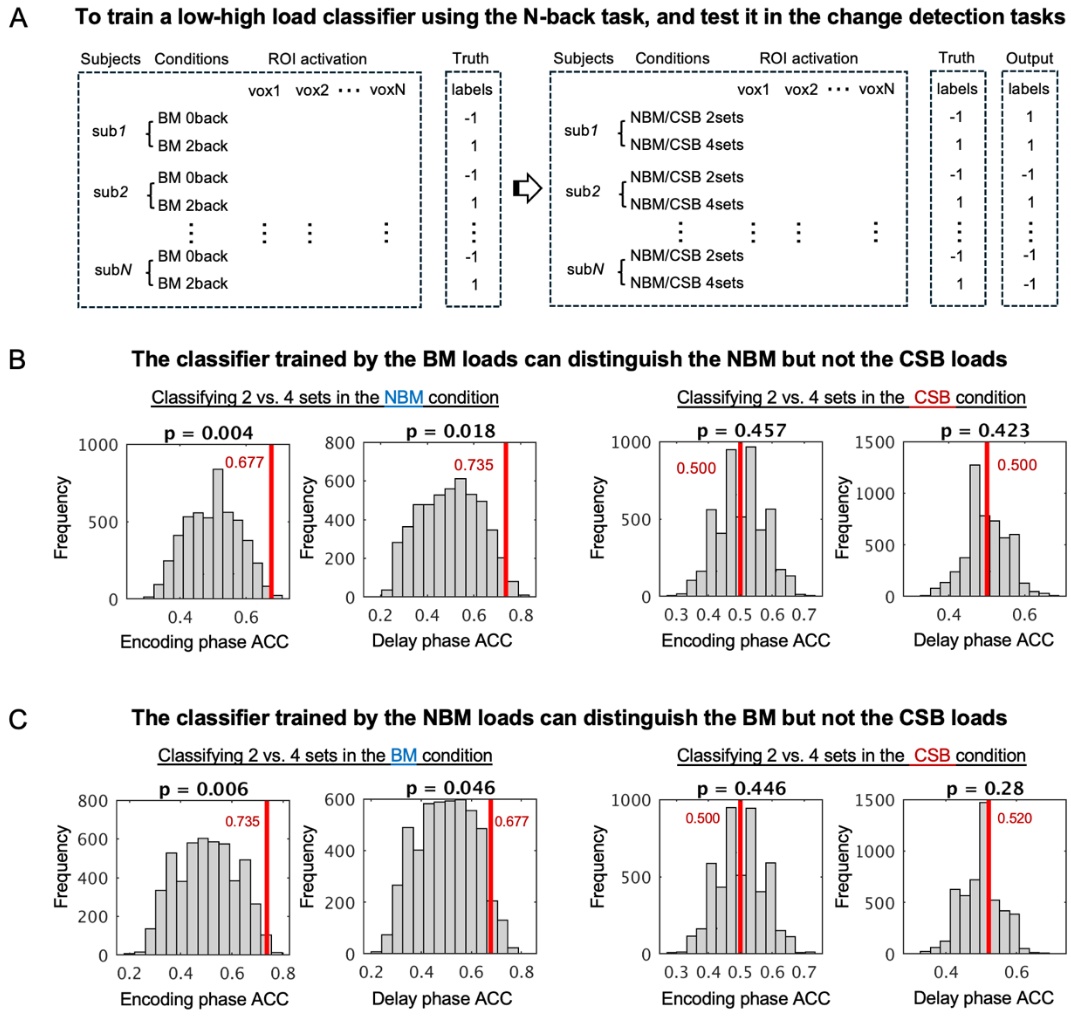

Fig. S14. Load classification performance. (A) Illustration of MVPA analysis for event load classification. (B) The model trained using activation from BM low load and BM high load in the N-back task can accurately classify NBM low load and high load in the event change detection task, but cannot classify CSB low and high load in the object change detection task. (C) The model trained using activation from NBM low load and NBM high load in the N-back task can accurately classify BM low load and high load in the event change detection task, but cannot classify CSB low load and high load. Note: number of permutations = 5000. Abbreviations: BM, biological movement; NBM, non-biological movement; CSB, color-shape binding; ACC, accuracy.

Fig. S15.

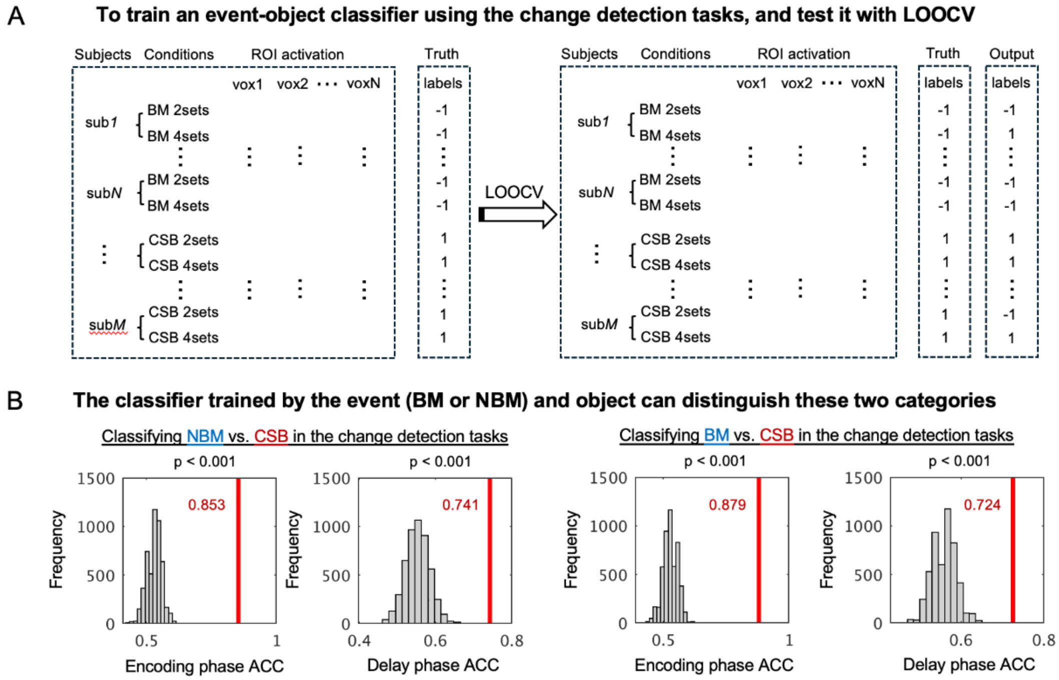

Fig. S15. Event and non-event classification performance. (A) Illustration of MVPA analysis for event and non-event classification. (B) The model trained using activation from BM (event) and object (non-event) conditions in the change detection tasks can accurately classify these two conditions, and this result was still significant when replacing BM with NBM. Note: number of permutations = 5000. Abbreviations: BM, biological movement; NBM, non-biological movement; CSB, color-shape binding; ACC, accuracy; LOOCV, leave-one-out cross validation.

Fig. S16.

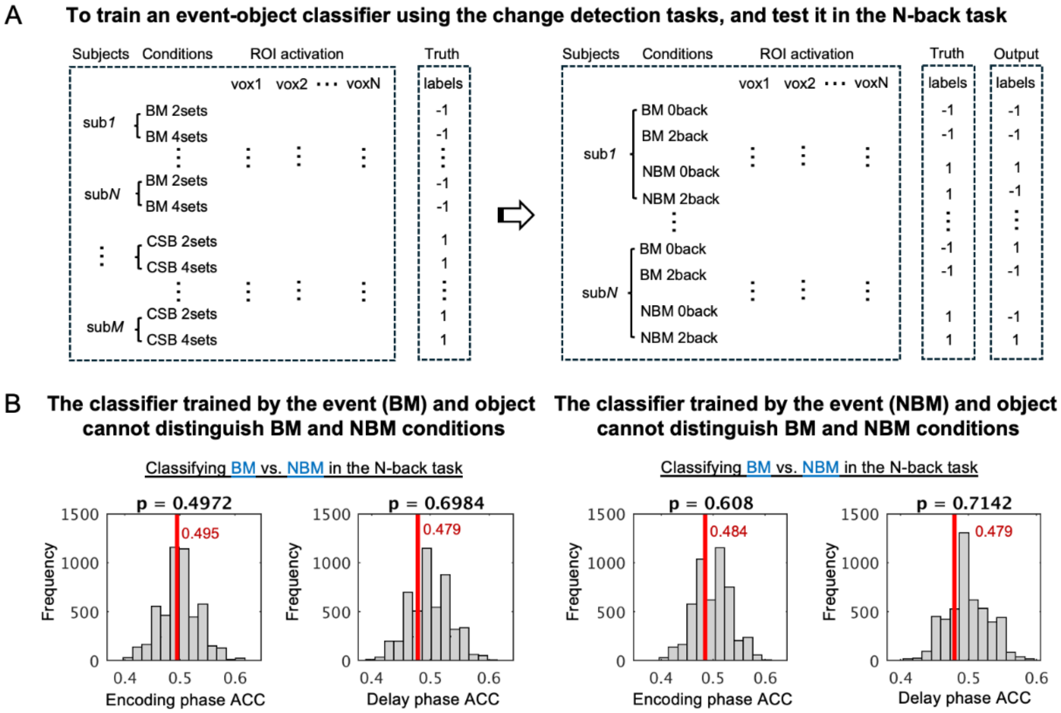

Fig. S16. Within-event category classification performance. (A) Illustration of MVPA analysis for BM and NBM classification. (B) The model trained using activation from BM (event) and CSB (non-event) conditions in the event/object change detection task cannot classify BM and NBM (both are events) in the N-back task, and same results were found when training a model using activation from NBM (event) and CSB (non-event) conditions in the event/object change detection task.

Supplementary Data Tables (Separate Files).

table S1. Descriptive statistics for WM tasks

table S2. Correlations among WM tasks

table S3. Fit statistics for working memory models

table S4. Evaluation of WM performance for different materials and conditions in dual tasks

table S5. Brain regions within each network

table S6. The relative degree of each network in prediction model

table S7. The most important nodes within the essential networks

table S8. Behavioral statistical analysis results for N-back, event change detection, and color-shape binding tasks

table S9. Statistics of ROI-based analysis

table S10. Statistics of timeseries analysis
